# “hDOS”: an automated hybrid diffuse optical device for real-time noninvasive tissue monitoring: precision and *in vivo* validation

**DOI:** 10.1117/1.JBO.30.11.115004

**Published:** 2025-11-18

**Authors:** Marta Zanoletti, Muhammad Atif Yaqub, Lorenzo Cortese, Mauro Buttafava, Jacqueline Martínez García, Caterina Amendola, Talyta Carteano, Lorenzo Frabasile, Diego Sanoja Garcia, Claudia Nunzia Guadagno, Tijl Houtbeckers, Umut Karadeniz, Michele Lacerenza, Marco Pagliazzi, Shahrzad Parsa, Tessa Wagenaar, Luc Demarteau, Jakub Tomanik, Alberto Tosi, Udo M. Weigel, Sanathana Konugolu Venkata Sekar, Alessandro Torricelli, Davide Contini, Jaume Mesquida, Turgut Durduran

**Affiliations:** a ICFO–Institut de Ciències Fotòniques, The Barcelona Institute of Science and Technology, Barcelona, Spain; b PIONIRS s.r.l., Milano, Italy; c Politecnico di Milano, Dipartimento di Fisica, Milano, Italy; d ASPHALION s.l., Barcelona, Spain; e BioPixS Ltd – Biophotonics Standards, IPIC, Tyndall National Institute, Cork, Ireland; f SPLENDO, Wassenaar, The Netherlands; g Hemophotonics s.l., Barcelona, Spain; h Politecnico di Milano, Dipartimento di Elettronica, Informazione e Bioingegneria, Milano, Italy; i Consiglio Nazionale delle Ricerche, Istituto di Fotonica e Nanotecnologie, Milano, Italy; j Fondazione IRCCS Ca’ Granda Ospedale Maggiore Policlinico, Milano, Italy; k Parc Taulí Hospital Universitari, Institut D’Investigació i Innovació Parc TaulÍ I3PT, Critical Care Department, Sabadell, Spain; l Institució Catalana de Recerca i Estudis Avançats (ICREA), Barcelona, Spain

**Keywords:** hybrid diffuse optics, time-domain near infrared spectroscopy, diffuse correlation spectroscopy, intensive care, standardization

## Abstract

**Significance:**

The hybrid diffuse optical system (hDOS) is a fully automated platform designed to bring advanced optical monitoring closer to clinical practice. Many existing systems lack automation, multiparametric output, and operator independence, limiting their use in demanding environments such as intensive care. hDOS integrates time-domain near-infrared spectroscopy, diffuse correlation spectroscopy, and pulse oximetry to assess both tissue oxygenation and perfusion. Although initially developed in the context of vascular occlusion tests, its modular design makes it suitable for broader applications, including trauma, surgery, anesthesia, and studies in healthy subjects.

**Aim:**

It aims to design and validate hDOS, focusing on precision, repeatability, and usability for peripheral microvascular monitoring in both clinical and research settings.

**Approach:**

Validation included test-retest measurements, a 7-month clinical deployment in the critical care, and a comparison with a commercial continuous wave NIRS device (INVOS 5100C).

**Results:**

The device underwent extensive validation, accumulating over 200 h of usage across ∼150 measurement sessions. The system showed high precision (test-retest CV <1.2% for oxygenation, <13% for perfusion), stable long-term performance, and lower variability than INVOS. It has also detected statistically significant differences during VOTs and detected hemodynamic impairment in ICU patients (n=100) compared with healthy volunteers (n=37).

**Conclusions:**

hDOS performed well in both bench and clinical settings, offering a unique combination of parameters in a fully automated, self-contained platform.

## 1 Introduction

Near-infrared spectroscopy (NIRS) is a noninvasive optical technique that utilizes light in the range of ∼650 to 950 nm to monitor tissue properties such as local microvascular blood oxygenation (${\mathrm{StO}}_{2}$) and blood volume. Over the years, NIRS has evolved as a promising tool for assessing tissue oxygenation and metabolism, with various advancements designed to overcome technological limitations.

Commercially available NIRS monitors predominantly employ continuous-wave NIRS (CW-NIRS), which is cost-effective and widely used in clinical settings. However, CW-NIRS lacks depth discrimination and cannot differentiate between changes in absorption and scattering (i.e., $\mu_{a}$ and $\mu_{s}^{\prime }$). As a result, it provides only relative measurements of hemoglobin concentration. Several adaptations of CW-NIRS, such as spatially resolved spectroscopy (SRS), broadband NIRS, and second derivative NIRS, have been proposed to improve the accuracy of tissue oxygenation indices.^1^^,^^2^ Despite these advancements, CW-NIRS faces challenges related to precision and reproducibility,^3^^–^^5^ making comparisons across studies difficult. Consequently, ${\mathrm{StO}}_{2}$ monitors have not achieved routine clinical implementation.

Frequency-domain NIRS (FD-NIRS) is another popular alternative, which uses intensity-modulated light (often radio-frequencies up to gigahertz range). FD-NIRS uses the physics of diffuse photon density waves to analyze the changes in their amplitude and phase to extract absolute values of both absorption and scattering coefficients. Over the years, with multiple modulation frequencies and multiple source-detector separations, FD-NIRS has emerged as a viable alternative to CW-NIRS.^6^ In our case, we have chosen to work with TD-NIRS as it contains even more information per source-detector pair, and the recently available instrumentation has greatly simplified TD-NIRS systems as reported here.

To address these limitations, time-domain NIRS (TD-NIRS) has emerged as a further alternative. Unlike CW-NIRS, TD-NIRS provides time-of-flight information that, in principle, not only allows a better discrimination of depth but also increases depth sensitivity.^7^^,^^8^ As in all diffuse optical methods, it can then be combined with more advanced algorithms and physical models to achieve better depth-resolved estimates. Most relevant to this paper is that it allows direct quantification of both absorption and scattering coefficients. This capability enhances the accuracy of assessing tissue oxygenation and perfusion. Historically, TD-NIRS systems were complex, bulky, and expensive, restricting their widespread adoption. However, advancements in laser sources, detection methods, and miniaturization have led to the development of compact and cost-effective TD-NIRS devices.^9^^–^^14^ The commercialization of TD-NIRS^10^^,^^15^ has further accelerated its potential for integration into clinical and research applications.

A significant advancement in optical monitoring has been the development of hybrid instruments that combine TD-NIRS with diffuse correlation spectroscopy (DCS). Although TD-NIRS provides depth-resolved information on tissue oxygenation by quantifying absorption and scattering properties, DCS measures microvascular blood flow through the analysis of dynamic light scattering.

This integration allows simultaneous measurements of tissue oxygenation and blood perfusion, offering a comprehensive assessment of microvascular function, addressing the limitation of traditional CW-NIRS. Hybrid NIRS-DCS systems have been applied in neuroscience and preclinical research as seen in the early studies on brain monitoring and animal models^16^^–^^20^ and it has been increasingly adopted in a wide range of clinical scenarios^21^^–^^30^

One prominent use case of NIRS is hemodynamic monitoring in the intensive care unit (ICU). As a noninvasive, bedside tool, NIRS assesses microvascular reactivity and endothelial function in critically ill patients, when combined with a vascular occlusion test (VOT).^31^ NIRS has been applied in ICU settings, including monitoring acute respiratory distress syndrome^32^ and COVID-19,^33^^,^^34^ weaning from mechanical ventilation,^35^^–^^40^ and evaluating microvascular reactivity in sepsis and septic shock.^41^^–^^52^ Beyond the ICU, NIRS has been utilized in trauma care,^53^^–^^57^ surgery,^58^^–^^61^ anesthesia,^46^^,^^62^^–^^65^ and severe medical conditions.^66^^–^^75^ NIRS has been utilized not only in the critically ill but also in advancing our understanding of microvascular function in healthy subjects^76^^–^^83^ and athletic performance.^84^^–^^88^

In this work, a detailed characterization and validation of the hybrid diffuse optical system (hDOS) device, developed during the VASCOVID project (European Union Horizon 2020 research and innovation, No. 101016087), is presented. The hDOS device is a hybrid optical multipurpose system designed for noninvasive, bedside assessment of microvascular oxygenation and blood perfusion in critically ill patients. The operation and use case of a former version of the hDOS device was previously described in Ref. 89

### 1.1 Objectives of the Device Validation

A holistic approach has been opted for in the evaluation of the hDOS device, going beyond the commonly reported “evaluation of basic performances” approach utilized for similar hybrid devices.^90^^–^^93^

**Fig. 1:**
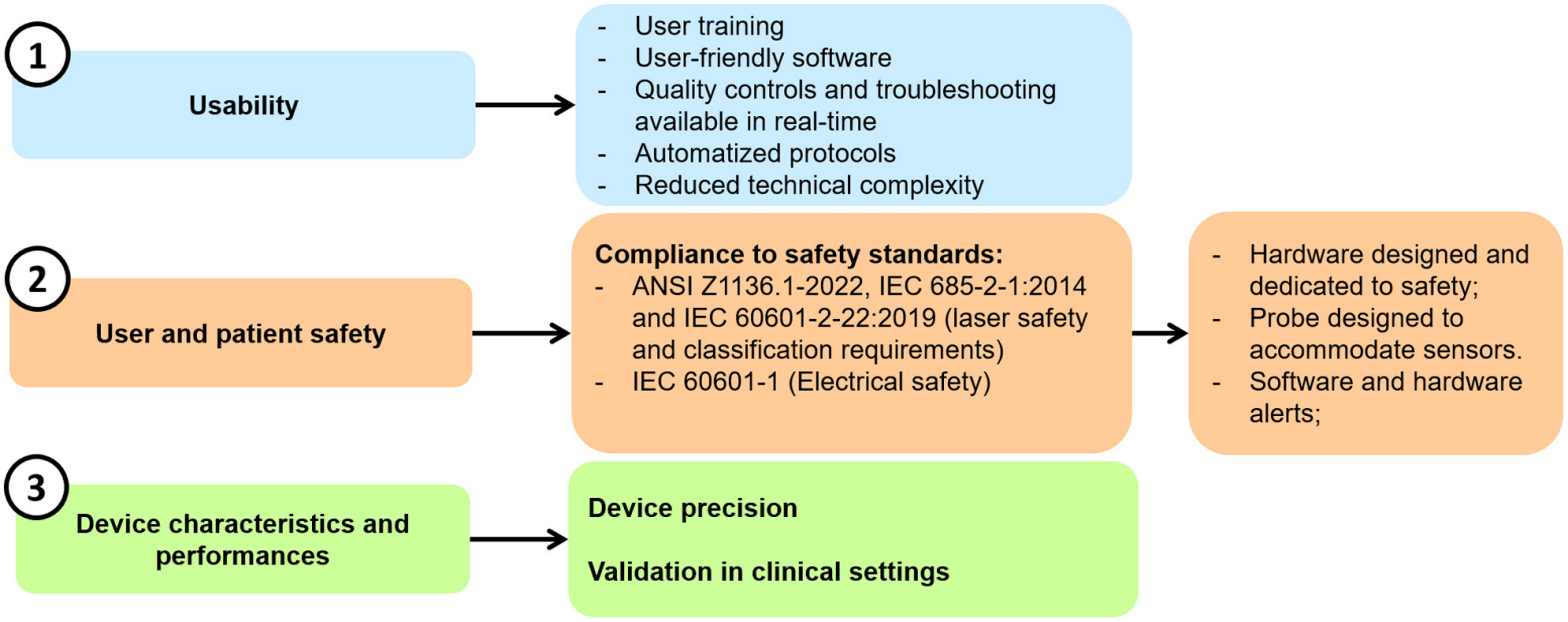
Illustrative overview of the key aspects that has been followed in the deployment of the hDOS device, along with the corresponding measures implemented.

The typical approach ensures that the basic optical performance metrics are met. In this case, a step further is taken by considering the replication of the systems and their independent operation in clinical settings. This is conceptually illustrated in Fig. 1, where the corresponding tests and measures implemented to address the problems and needs of the end-user are outlined.

Detailed descriptions of how these measures were integrated during the design phase are provided in Sec. 2.1 and their use in a typical protocol is described in Ref. 89.

Three key aspects have been addressed in the design and development of the hDOS device: (1) usability, (2) user and patient safety, and (3) device characteristics and performances.

A typical research system developed by us and other laboratories^22^^,^^90^^,^^91^^,^^94^^–^^97^ rely on extensive training and the experience of the operator for clinical study deployment. Here, the system was provided with user-friendly software for independent clinical use with minimal training. Extensive automated safety checks were implemented, detailed data and probe placement metrics were used to provide front panel and on-screen alerts, and software messages were designed to guide users in understanding the signal, identifying problems, and troubleshooting. The software guides users through the protocol and provides real-time access to both raw and processed data, thereby reducing the complexity of operating the device.

Real-time safety measures for both operators and patients have been incorporated through hardware and software tools. Continuous monitoring and quick-response mechanisms are used to maintain safe operation. Quality indicators are also included to alert when the laser is active, environmental light interferes with the signal, or real-time data fitting is not optimal, as described in Sec. 2.

The device has been deployed in the ICU at Parc Taulí Hospital Universitari for seven months. This evaluation was conducted not only for usability and training purposes but also to assess the device performance in the ICU environment. Tests have been conducted on both tissue-mimicking phantoms and *in vivo* to test the device’s precision and to set the basic performances in clinical settings. In particular, the device performance during probe repositioning (test-retest) and its susceptibility to interference affecting the two optical modules (as detailed in the dedicated section) were evaluated, and comparisons were made with a commercially available NIRS device used in the clinics.

## 2 Methods

### 2.1 Platform Description

**Fig. 2:**
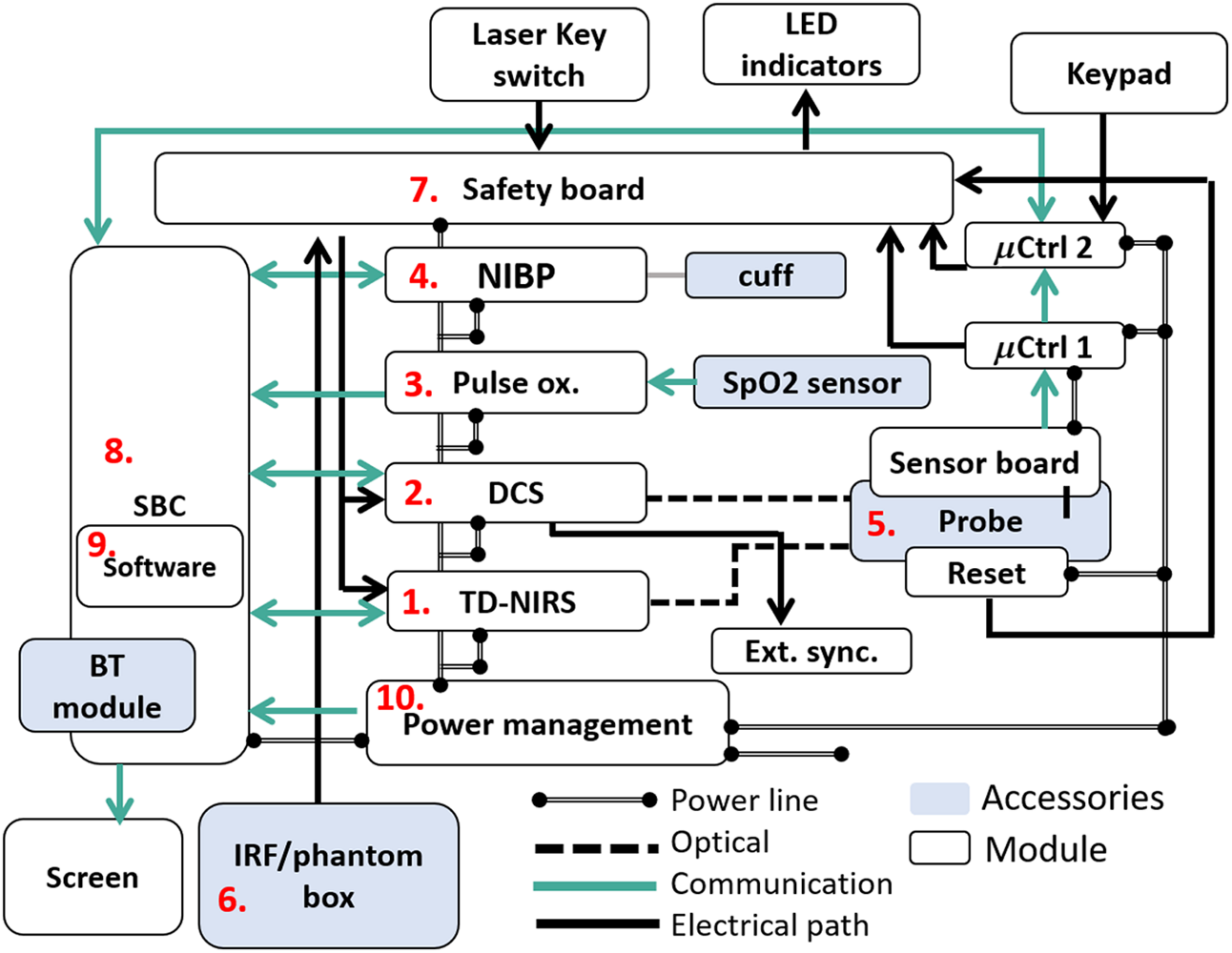
Schematic of the device. (1) TD-NIRS module; (2) DCS module; (3) pulse oximeter module connected to the ${\mathrm{SpO}}_{2}$ sensor; (4) noninvasive blood pressure module (NIBP) connected to the properly sized cuff; (5) optical probe and sensor board communicating with two microcontrollers (μCtrl 1 and 2); (6) IRF/phantom box; (7) safety board and primary connection with the other primary modules; (8) single board computer with (9) in-house made software running onboard. A Bluetooth module is available on board for remote control. The SBC is also connected with an internal and/or external screen; (10) power management system. TD-NIRS, time-domain near-infrared spectroscopy; DCS, diffuse correlation spectroscopy; Pulse ox., pulse oximeter; 4. NIBP, noninvasive blood pressure; μCtrl 1 and 2, micro-controllers 1 and 2; IRF, instrument response function; SBC, single board computer (SBC).

**Fig. 3:**
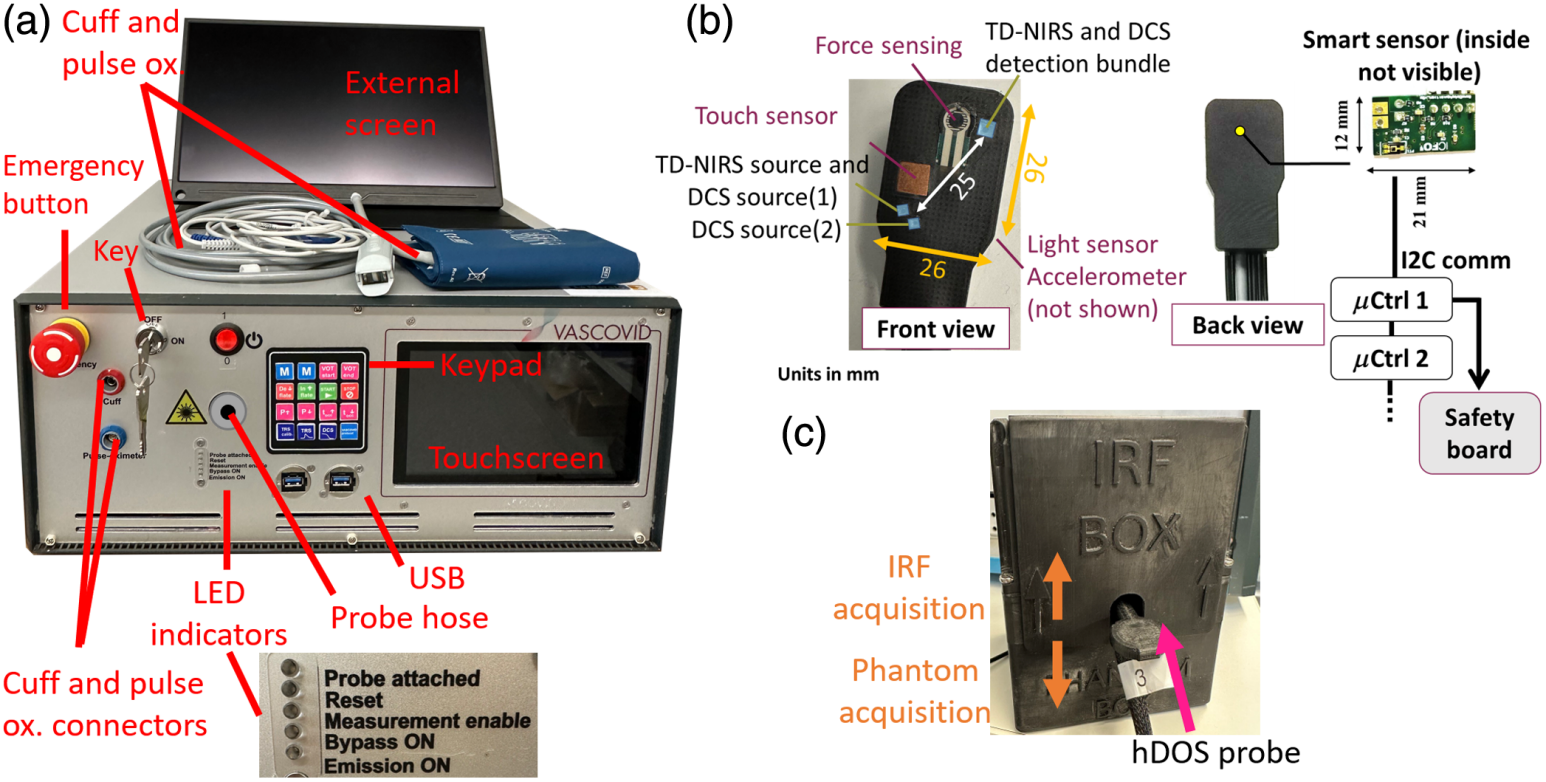
Picture of the hDOS device with its accessories; (a) whole platform with main components and a zoom on the LED indicators; (b) front and back views of the optical probe together with its smart sensor board embedded within the probe; (c) IRF/phantom box.

This platform was developed in a close-knit collaboration in a modular fashion based on the experience accumulated by several partners involved in this project and is illustrated in Fig. 2. Here, we highlight the primary links between the modules and their communication paths to ensure proper operation and include a picture of the device and its accessories in Fig. 3.^89^

The following paragraphs will detail each module, as indicated in the figure, explaining the design choices and features. A thorough description of the use of a previous (yet very similar) version of this device has been published elsewhere.^89^ Here, we focus on salient technical details and features that define the hDOS device.

#### 2.1.1 Time-domain NIRS, TD-NIRS

The TD-NIRS module (1) is an original equipment manufacturer (OEM) customized version of the NIRSBOX (PIONIRS s.r.l., Italy).^14^ TD-NIRS^9^^,^^98^^,^^99^ employs two pulsed lasers working at 53 MHz, emitting short pulses at 685 and 828 nm (with the duration in the order of ≈100  ps) that are shone into the tissue by means of a bifurcated bundle composed of two 100/140  μm glass graded index multimode fibers (NA = 0.22). The lasers are also coupled with optical attenuators, which are commanded via software to reach the correct signal-to-noise ratio (SNR). Laser pulses from each wavelength are injected alternatively into the bundle.

The maximum power injected into the tissue is 3.5 and 3.8 mW for 685 and 828 nm, respectively, and it is limited by the maximum permissible emission by the ANSI Z1136.1-2022 and IEC 6825-2-1:2014.^100^^,^^101^

Furthermore, the TD-NIRS module is equipped with an interlock system that receives a transistor-to-transistor logic (TTL) signal that originates from the safety board (7), and it allows for controlling the TD-NIRS laser emission. Diffuse light is then recollected by a 1 mm graded index core plastic multimode fiber (NA = 0.39) placed at 2.5 cm from the source, which is in turn fed into a silicon photomultiplier detector. Wavelength selection is made by a custom-made dual pass band filter (in-band transmissivity >90%, out-band rejection >4 OD). Both source and detector fibers are hosted in the optical probe (5).

The distribution of time of flight (DTOF) of photons is reconstructed at each wavelength every second, with an integration time of 500 ms, using a custom digital single photon counting unit.

The TD-NIRS module communicates with a single board computer (SBC) (8) to exchange data and time stamps, as well as other important information such as status from the lasers and other internal controls. Primary communication is via USB 2.0. All the information saved and exchanged with this module are summarized in Table S1, available in the Supplementary Material.

#### 2.1.2 Diffuse correlation spectroscopy, DCS

DCS (custom developed based on ICFO/HemoPhotonics S.L., Spain modules)^16^^,^^102^^,^^103^ (2) utilizes long-coherence length (>10  m), CW light at 785 nm to quantify the statistics of the diffuse laser speckles fluctuations as impressed by the movement of red blood cells.^102^^,^^104^

Light at 785 nm is coupled into a fiber splitter (200  μm core and NA = 0.22) and then fed into two step index 400  μm core (NA = 0.39) fibers. For this particular case, CW light at 785 nm is limited to 28 mW by the ANSI Z1136.1-2022 and IEC 6825-2-1:2014.^100^^,^^101^

To comply with these limits while maximizing SNR, the injection is split into two injection points located along an arc of 3.5 mm, centered at the same source-detector distance (2.5 cm) used for DCS. This dual-source configuration allows approximately the same tissue volume to be probed while spatially distributing the optical power. This arrangement doubles the detected intensity and improves SNR while staying within international standards for safety.^90^^,^^105^^,^^106^

CW light is then collected by two 4.4/125  μm core/cladding diameter single-mode (NA = 0.13) fibers. As TD-NIRS and DCS measurements are performed simultaneously, each DCS detection fiber is connected to a pigtailed bandpass filter (OZ optics Ltd., Canada).

Finally, the detection fibers are fed each into a single photon avalanche photo diode whose TTL output is utilized by a hardware correlator to construct the intensity autocorrelation function $g_{2}(\tau )=\langle I(t)\cdot I(t+\tau )\rangle /{\left\langle I(\tau )\right\rangle }^{2}$, where t is the measurement time, τ is the lag time and ⟨·⟩ denotes a time average. In this case, DCS quantifies the $g_{2}$ over an averaging time of 26 ms, allowing the resolution of the pulsatility of blood flow due to the cardiac cycle.

The DCS acquisition is triggered by the same TTL signal, generated by the safety board (7), which also initiates the TD-NIRS acquisition. Once DCS starts acquiring data, it continuously acquires and returns data at the rate specified by the user. An additional TTL signal (1 to 100 Hz) is available for external connections to synchronize with other clinical monitors and/or data aggregation platforms (Ext. sync.).

Finally, the DCS module communicates to the SBC (8) via USB 2.0, to exchange data collected ($g_{2}$, intensity and time stamps). All the information saved and exchanged with this module are summarized in Table S2, available in the Supplementary Material.

#### 2.1.3 Pulse oximeter, ${\mathrm{SpO}}_{2}$ module

The platform features a pulse oximeter (Medlab GmbH, Germany) (3) that continuously and synchronously calculates the photoplethysmograph signal at 50 Hz (PPG), arterial oxygenation (${\mathrm{SpO}}_{2}$), heart rate (HR), and perfusion index at 1 Hz. The module communicates with the SBC (8) via serial communication RS-232. All the information saved and exchanged with this module are summarized in Tables S3 and S4 available in the Supplementary Material.

#### 2.1.4 Automated tourniquet, NIBP module

The automated tourniquet (custom-developed by Medlab GmbH, Germany) (4) has been modified to support rapid inflation and deflation rates, recommended for VOT. Cuff pressure data are transmitted to the SBC (8) at 5 Hz, with a maximum recommended inflation pressure of 300 mmHg. The device includes a range of cuff sizes (Medlab GmbH, Germany) designed to accommodate different limb circumferences. These cuffs are fabricated from biocompatible flexible polyurethane and can be reused following disinfection.

This module communicates with the computer via RS232 serial communication. All the information saved and exchanged with this module are summarized in Table S3 available in the Supplementary Material.

#### 2.1.5 Probe

The primary objectives in designing the multimodal probe were to ensure comfort, safety, and data quality. In this paragraph, the rationale and methods behind achieving these goals will be explained. A picture of the probe is shown in Fig. 3(b).

TD-NIRS and DCS fibers are encased in a durable, 3D-printed black rubber material with a shore hardness rating of 85A. The sources are organized such that one right-angle 2 mm prism couples the TD-NIRS bifurcated bundle and one DCS source to the tissue, whereas a second 2 mm prism, positioned 3.5 mm away, delivers light from the second DCS source, forming the dual-injection configuration. For detection, all the TD-NIRS and DCS fibers are combined into a single bundle, which is then coupled with the tissue by using a right-angle 3 mm prism. Each module source and detector pairs are 2.5 cm far from each other. Due to the geometrical arrangement, filters have been positioned in front of the TD-NIRS and DCS to accurately select the wavelengths of interest, as explained in the previous sections.

Each fiber is enclosed in a lightweight vinyl sleeve with a minimal diameter and all are ultimately protected by a meshed sleeve. The total length of the probe is 3 m, which ensures minimal losses for the fibers utilized for the TD-NIRS systems at both wavelengths.

The arrangement of the source and detector is designed to accommodate a touch sensor (7×7  mm×0.02 inch copper plate) for detecting contact, as well as a force-sensing resistor (FSR400) for monitoring pressure changes exerted by the probe on the tissue.

Both capacitive and force sensor terminations are connected through coaxial cables to their respective sensing chip and feedback circuit embedded on a custom printed circuit board placed on the probe head.

In addition to a contact and force sensor, a 3-axis accelerometer and a photodiode have been included.

On the back of the probe head, a smart sensor board (12×21  mm) is enclosed, which transmits data to a microcontroller (μCtrl 1) via I2C communication protocols. The μCtrl 1, based on a pre-set threshold on the touch sensor, sends a TTL signal to the safety board (7) to indicate the probe attachment to the tissue. The μCtrl 1 also transmits data to a second microcontroller (μCtrl 2), which then communicates with the SBC (8). The data received from μCtrl 2 are utilized by the software to set user and device alerts. The use of two sequential microcontrollers is aimed at minimizing the possibility of accessing the sensor board and overriding the safety signals.

Due to the presence of signal and power lines, an electrical isolator is positioned along the probe to decouple the patient from the device. In addition, a reset switch has been implemented, which must be pressed if probe detachment is detected for more than 10 s, in compliance with the latest safety standard IEC 60601-2-22:2019.^107^ If the switch is not pressed, laser emission is not enabled. Collectively, these and other measures (see below) taken allow this device to be characterized as being in Laser Class 1C as opposed to Class 3B.

Alerts and indicators of signal quality are available to the user and for postprocessing as shown in Table S3, available in the Supplementary Material.

#### 2.1.6 IRF/phantom box

The platform is equipped with a smart box that facilitates daily instrument response function (IRF) assessment,^108^ which also includes a durable, solid tissue-simulating phantom (BioPixS Limited, Ireland) for TD-NIRS quality control. The smart IRF/phantom box [Fig. 3(c)] is equipped with an electrical circuit made of strategically placed mechanical switches. When the probe is inserted into the box, these switches are activated, generating a signal that is fed to the on-board safety control unit, which in turn alerts the user by activating the “probe attached” LED indicator on the front panel and engages the internal interlocks for the TD-NIRS and DCS lasers. This feature is designed to simulate the attachment to tissue via a contact sensor, which does not function with the phantom. Once the “attachment” is detected, the operator is allowed to acquire an IRF and/or a phantom measurement. The IRF measurement involves acquiring a single DTOF for 1 s, aiming for a photon count rate of $10^{6}$ photons per second for each wavelength. The key IRF parameters, such as the temporal position of its barycenter and full-width-half-maximum (FWHM), are calculated real time by the on board software (9). In particular, the FWHM represents the temporal resolution, whereas its barycenter reflects the stability of the laser, detection, and acquisition chain, which ultimately impacts the retrieval of the tissue’s optical properties.

The phantom utilized in this case is made of silicone background, with absorption contribution given by carbon black and scattering given by titanium dioxide, with desired optical properties at 685 nm $\mu_{a}=0.23\;{\mathrm{cm}}^{-1}$ ($\mu_{s}^{\prime }=12.8\;{\mathrm{cm}}^{-1}$) and at 828 nm $\mu_{a}=0.19\;{\mathrm{cm}}^{-1}$ ($\mu_{s}^{\prime }=9.5\;{\mathrm{cm}}^{-1}$) (Biopixs, s.l., Ireland). The phantom measurement in particular is made of 20 repetitions with acquisition time of 1 s and target count rate of $10^{6}$ counts per second per wavelength. Also, for the phantom measurements the on board software (9) calculates in real time the FWHM, the barycenter as well as the optical properties of the phantom.

Throughout the entire validation process, all users, including lab technicians and clinicians, have been guided to acquire an IRF to accurately calibrate TD-NIRS measurements as well as a phantom measurement at each session. The purpose is to assess the day-to-day performance in terms of precision and reproducibility, as well as quantifying potential degradation of the module over time.

#### 2.1.7 Safety board

To integrate and manage all the safety features from the previously described modules, a dedicated safety board featuring key components such as μCtrl 1 and 2 has been implemented. The safety board operates by generating the laser-on signal for both TD-NIRS and DCS based on several conditions:

• the key on the front panel must be in the “on” position;
• the user reset button on the probe must be “off”, indicating that the probe is attached and no detachment (monitored by μCtrl 1) longer than 10 s has occurred;
• μCtrl 2 must receive the software command to enable the lasers for both systems, subsequently communicating this to the safety board;
• while using the IRF/phantom box the probe must be inserted properly for the “attachement” to be sensed.

If any of these conditions is not fulfilled, the safety board activates/deactivates LEDs placed on the front panel to indicate the necessary action that the user needs to take. This ensures prompt attention to maintain operational safety.

#### 2.1.8 Single board computer

The platform is controlled by an industrial-grade single board computer (SBC-230D N4200, ASRock Industrial Computer Corp. Taiwan), which includes a built-in touchscreen and keypad. The keypad is programmed for performing manual markings, initiating pre-programmed protocols, and managing initial calibration or self-test procedures. It connects to the μCtrl 2, which communicates any keypress to the SBC. The SBC handles critical functions such as running the software, managing communication with all modules for sending commands and receiving data, and ensuring reliable data storage.

In addition, remote operation is enabled through Bluetooth technology. This feature is particularly useful in settings such as infectious disease triage.

#### 2.1.9 Software

The onboard software is tailored to meet the needs of medical teams. It provides real-time plots of oxy-, deoxy- and total hemoglobin (HbO, HbR and tHb) concentrations, ${\mathrm{StO}}_{2}$, blood flow index (BFI) and quality parameters for TD-NIRS, DCS, and pulse oximeter readings. How these parameters are extracted in real time has already been described in Ref. 89.

Operators can also access a device monitor displaying intensity autocorrelation functions for DCS and DTOFs for the TD-NIRS module.

**Fig. 4:**
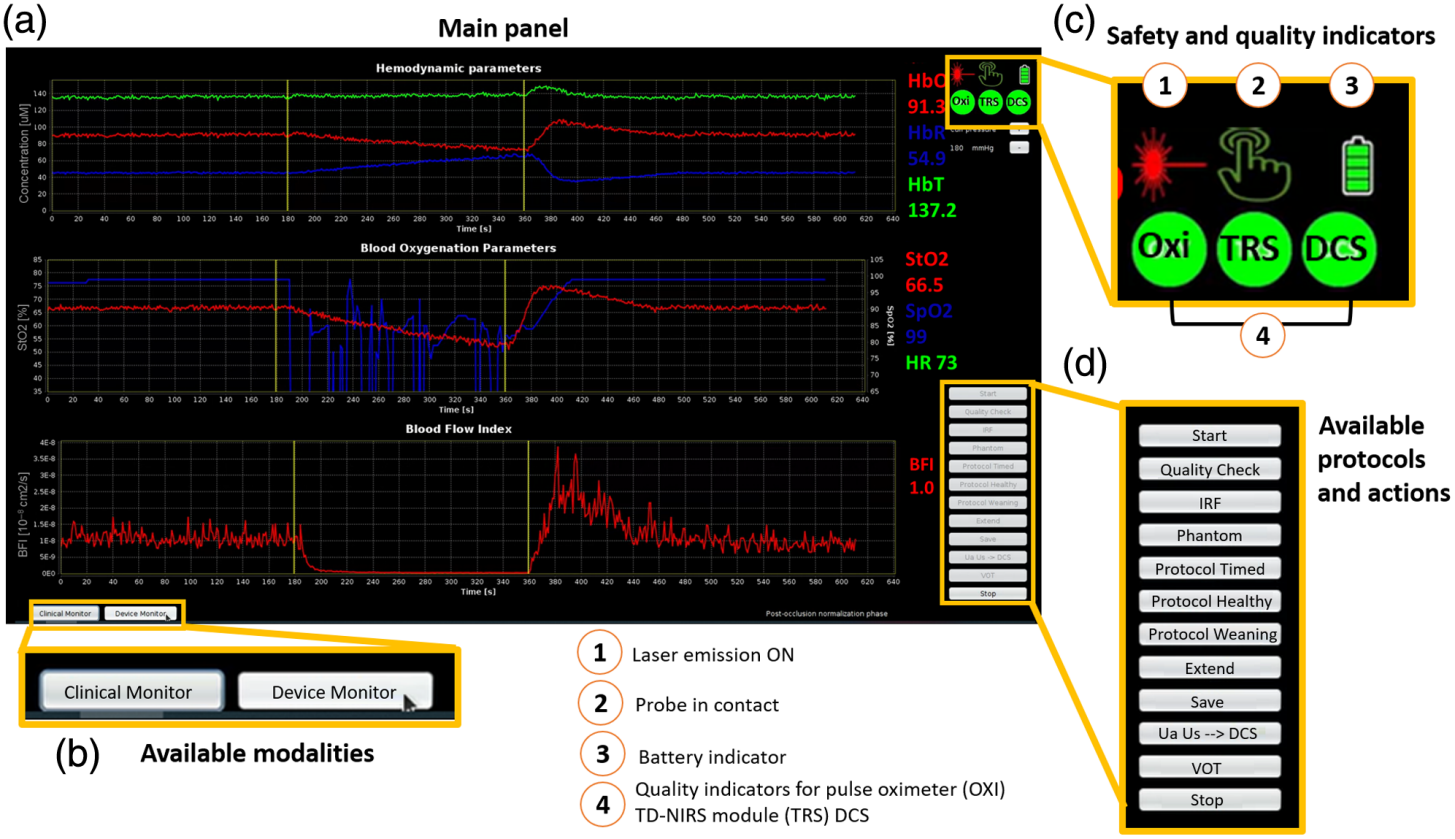
Snapshot of the hDOS device user interface: (a) the main panel displays real-time parameters such as hemoglobin concentrations (HbO, HbR, and tHb in μM), ${\mathrm{StO}}_{2}$, ${\mathrm{SpO}}_{2}$ (in %), and BFI (in ${\mathrm{cm}}^{2}/s$; (b) the available modalities include a clinical monitor (main panel) for parameter display and a device monitor for raw data quality assessment; (c) real-time safety and quality indicators for ${\mathrm{SpO}}_{2}$ (OXI), TD-NIRS, and DCS (labeled as TRS and DCS, respectively) are provided. A battery indicator is also included as the device operates on battery power; (d) available protocols. HbO, oxygenated hemoglobin; HbR, deoxygenated hemoglobin; tHb, total hemoglobin; ${\mathrm{StO}}_{2}$, tissue oxygen saturation; ${\mathrm{SpO}}_{2}$, peripheral oxygen saturation; and BFI, blood flow index.

The software suite comprises two applications: one developed in NetBeans IDE 8.2 using Java Development Kit 8 and another in Python 3.11 for Bluetooth communication. Both applications run on the Ubuntu 22.04 LTS operating system. The hDOS device software’s main panel displays time traces and measurements in real-time [Fig. 4a)]. Users can also monitor safety and quality indicators, such as emission and probe attachment status, to ensure optimal device performance [Fig. 4(c)]. Different actions are available to the operator. Details about the software and the different protocols available are already explained elsewhere alongside their functions.^89^

#### 2.1.10 Power management system

The power management is handled by a dedicated board designed to convert and distribute the necessary power from four battery packs, each connected to its respective power management system (RRC Power Solutions GmbH, Germany). These power management systems can seamlessly switch between battery and external power supply. In addition, the batteries communicate their charge status and other critical information to μCtrl 2, ensuring efficient power monitoring and management. The addition of a power management module ensures the device’s usability in challenging settings, such as ICU, where numerous monitors are used simultaneously, and power plugs are not always readily available.

Finally, the device has been tested by means of an electrical safety analyzer, which is compliant with the IEC 6060-1 standard.^109^ An emergency button has been incorporated and strategically placed to be easily accessible on the front panel, allowing for quick action. This safety feature ensures that all the electrical lines are disconnected, thereby stopping the device’s operation and preventing potential harm to the operator and patients.

### 2.2 Analysis Method for TD-NIRS and DCS, and VOT-Derived Biomarkers

The retrieval of the parameters of interest in real time are explained in Ref. 89.

For DCS, the $g_{2}(\tau )$ functions acquired every 26 ms were block-averaged *a posteriori* to 1 Hz to match the TD-NIRS acquisition rate. A 1 Hz rate was chosen as a compromise: it reduces noise while maintaining sufficient temporal resolution to capture hemodynamic responses during VOT, although cardiac pulsatility (∼1 to 1.5 Hz) is not resolved and instead contributes to the variability in the BFI estimates. The VOT-derived parameters were calculated using a semi-automated script developed in MATLAB2021b (The MathWorks Inc., Natick, Massachusetts, USA). Different approaches are possible according to the needs of a specific study.

#### 2.2.1 VOT-derived parameter calculation

**Fig. 5:**
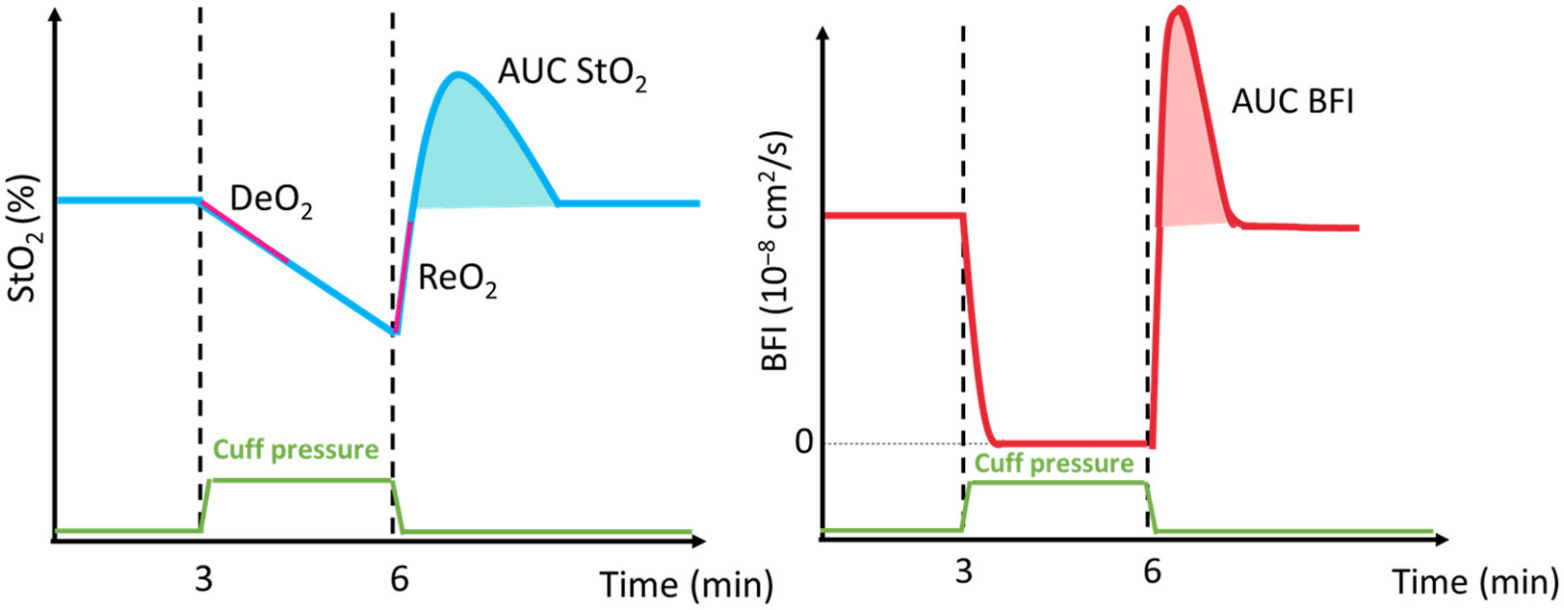
Schematic response of a VOT for ${\mathrm{StO}}_{2}$ (%) and BFI (${\mathrm{cm}}^{2}/s$). Black vertical dashed lines represent the inflation and the deflation points. The purple lines represent (i) the linear fitting of the first minute of occlusion with its slope being the ${\mathrm{DeO}}_{2}$ (%/min) and (ii) the linear fitting upon the cuff release, with its slope being the ${\mathrm{ReO}}_{2}$ (%/s). The shaded areas represent under the curve in both ${\mathrm{StO}}_{2}$ (%·min) and BFI (${\mathrm{cm}}^{2}$).

Fig. 5 reports a schematic of the typical response to an ischemic challenge for the ${\mathrm{StO}}_{2}$ and BFI parameters. The VOT corresponds to an initial 3 min baseline, a 3 min arterial occlusion to a pressure exceeding the systolic pressure by 50 mmHg, and a recovery period of 5 min upon cuff release.

The VOT-derived parameters are also highlighted: i) baseline phase for both ${\mathrm{StO}}_{2}$ and BFI, ii) deoxygenation rate (${\mathrm{DeO}}_{2}$) corresponds to the slope of the linear fitting of the first minute of occlusion, iii) reoxygenation rate (${\mathrm{ReO}}_{2}$) is the slope of the linear fitting from the deflation point (corresponding to the minimum ${\mathrm{StO}}_{2}$ reached during the occlusion) to the intersect of ${\mathrm{StO}}_{2}$ with the second baseline reached in the recovery period and iv) the area under the curve (AUC) ${\mathrm{StO}}_{2}$ and AUC BFI is the area calculated from the first to the second intersect of ${\mathrm{StO}}_{2}$ with the baseline reached in the recovery period. If parameters do not return to baseline within the standard 5-min recovery period, the system allows the user to extend the acquisition with a button press at 30 s increments. In cases where recovery is still incomplete, baseline-dependent metrics such as AUC (${\mathrm{StO}}_{2}$ and BFI) and ${\mathrm{ReO}}_{2}$ are considered unreliable and are excluded from the analysis.

#### 2.2.2 Baseline metabolism assessment

The index of local metabolic rate of oxygen extraction for the skeletal muscle (${\mathrm{MMRO}}_{2}$) is derived at the baseline by the simultaneous and independent assessment of BFI (oxygen transport to the tissue) along with arterial and microvascular tissue oxygenation (oxygen utilization), without the need for a vascular occlusion. In a steady-state condition, ${\mathrm{MMRO}}_{2}$ can be calculated as ${\mathrm{MMRO}}_{2}\approx [\mathrm{Hb}]\cdot ({\mathrm{SpO}}_{2}-{\mathrm{StO}}_{2})/\gamma {\mathrm{SpO}}_{2}\cdot \mathrm{BFI}$, as derived from the Fick’s law,^16^^,^^19^^,^^110^ where [Hb] is the hemoglobin concentration in g/dL and γ is the percentage of blood content in the venous compartment.

### 2.3 Precision of the hDOS Device

The hDOS device validation has been performed mainly with a focus on the optical modules. The accessory modules, such as the pulse oximeter and the automatized cuff, are tested separately by the OEM according to their standard operating procedures. This section focuses on the tests conducted to evaluate the precision of the device, specifically in four areas: (i) variability of key parameters *in vivo* and on phantom at module level; (ii) the variability observed during probe placement and repositioning, and (iii) the device variability under different detected light levels and finally, (iv) optical interferences during measurements.

The following paragraphs will provide an in-depth explanation of the protocols used to address these aspects.

#### 2.3.1 Variability of key parameters at module level

As explained in Sec. 2.1.6, all users were instructed to acquire an IRF and a phantom measurement prior to each session. For each phantom the effective ${\mathrm{tHb}}^{\mathrm{ph}}$ and ${\mathrm{StO}}_{2}^{\mathrm{ph}}$ were computed, using the hemoglobin absorption coefficients at 685 and 828 nm, similar to a previous study.^111^

The coefficient of variation (CV) is a typical figure of merit for the assessment of day-by-day variability and it is defined as the ratio between standard deviation(x) and the mean(x) (in %) over the entire clinical study, where x is the measurand of interest, such as FWHM, barycenter, optical properties and effective ${\mathrm{tHb}}^{\mathrm{ph}}$ and ${\mathrm{StO}}_{2}^{\mathrm{ph}}$. To simplify the calibration process and improve the overall efficiency of the system, as a proof-of-concept, the first IRF acquired in month 3 was utilized to process all the phantom measurements acquired in that same month. It has then been compared with the standard day-by-day calibration. This comparison was performed using the Wilcoxon signed-rank test with a significance level set at p<0.05.

For DCS, robust quality control cannot be performed using a solid phantom, as it only provides fixed scattering properties and does not reproduce the dynamic changes needed to assess flow or motion-related metrics. To keep the measurement workflow practical and efficient, liquid phantoms were not included in the routine quality checks for each session.

On the other hand, the raw $g_{2}$ is carefully analyzed at each *in vivo* measurement session. The count rate and the β can be used as quality parameters. The β parameter is a constant number related to the number of modes detected. In a typical DCS device, β≈0.5 and it has to remain stable throughout the whole measurement time. A drop in this parameter might be due to instabilities of the laser, noncoherent light leakage, or probe detachment. To test the variability of the DCS module, the CV over the count rate and the β are reported during the baseline. For β the median and the standard deviation are calculated from 60 s of baseline measurements where the raw $g_{2}$ is integrated by 10 s. In the same 60 s of baseline, the median and standard deviation of the count rate is obtained in kHz. To determining if specific trends are visibile, Spearman’s correlation analyses were conducted for both β and count rate. In addition, constant user feedback helped us in identifying fiber issues and power losses, which were promptly addressed.

#### 2.3.2 Test-retest variability

To assess the variability due to probe repositioning, a test-retest experiment has been performed on the *brachioradialis* muscle of one subject by repositioning the probe five times and acquiring data for about 200 s at 1 Hz for both DCS and TD-NIRS, similar to what is reported in other works by us and others.^14^^,^^111^ Finally, the mean values of ${\mathrm{StO}}_{2}$ as well as BFI in the five tests and as well as the intra-test and inter-test variability as CV, have been calculated.

#### 2.3.3 Dependence of precision with respect to absolute values

A custom protocol was implemented to assess the device precision and identify small but meaningful alterations in both ${\mathrm{StO}}_{2}$ and BFI. This was done by occluding arterial blood flow at increments of 40%, 60%, 80%, and 100% of the limb occlusion pressure (LOP) during both inflation and deflation. The LOP was identified as the point at which the PPG signal vanishes during limb occlusion. For each occlusion interval, lasting 60 s, the CV was calculated for both BFI and ${\mathrm{StO}}_{2}$, over the last 15 s of the inflation or deflation interval.

**Fig. 6:**
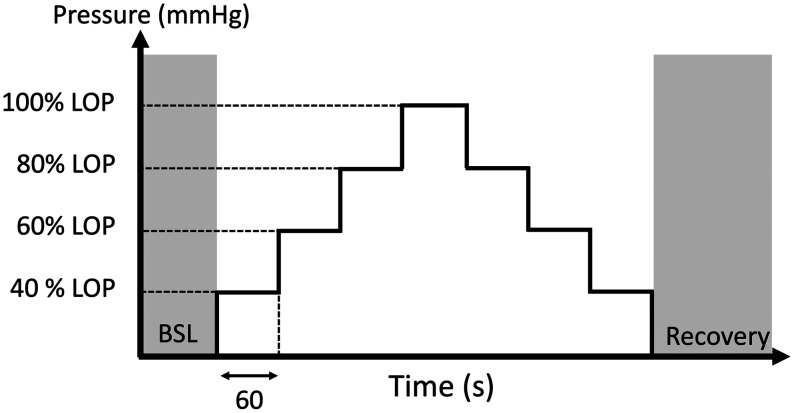
Schematic of the protocol for the assessment of the sensitivity of the platform *in vivo*. The cuff is inflated and deflated every 60 s at pressures equal to 40%, 60%, 80%, and 100% of the LOP. LOP, limb occlusion pressure; BSL, baseline.

The protocol is shown in Fig. 6. The dependence of the CV on the mean absolute values calculated across the different inflation/deflation periods was then analyzed, along with how CV changes at the various signal levels, as indicated by the count rate (for DCS) or dynamic range (for TD-NIRS), by means of Spearman’s correlation. The dynamic range is calculated as the ratio of the maximum of the DTOF (in number of photons) over the standard deviation of the background.

#### 2.3.4 Optical interference

**Fig. 7:**
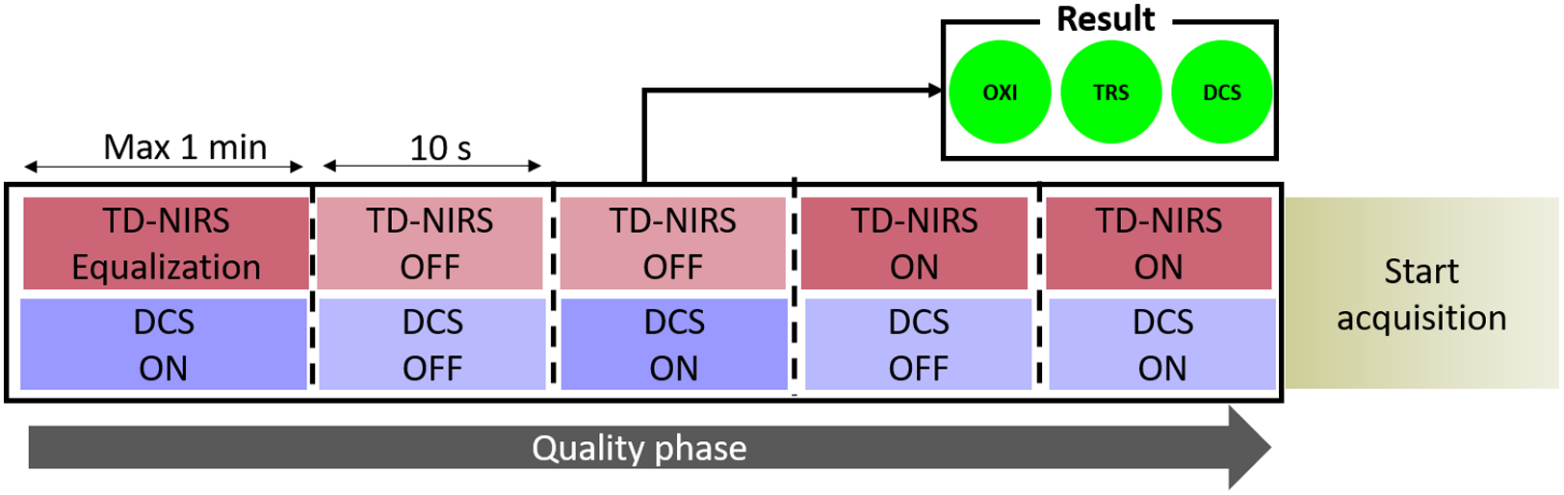
Schematic of data QP. After an the inital equalization of the TD-NIRS, lasers are switched ON and OFF alternatively for a time interval of 10 s. The first minute correspond to the equalization phase of the TD-NIRS lasers.

A specific “quality assessment phase” (QP) has been implemented to continuously assess the quality of TD-NIRS, DCS, and pulse oximeter signals throughout the protocol. During this phase, the internal software alternates between switching DCS and TD-NIRS sources on and off to evaluate potential interference and dark light levels. This phase begins with signal equalization for TD-NIRS up to $10^{6}\;\text{counts}/s$ and adjusts DCS signals to the maximum deliverable power. The QP lasts less than 2 min, and its results are stored in data files and displayed to the user via quality indicators that turn red or green based on the outcome (Fig. 7). The outcome is summarized by three front-panel indicators (TRS, DCS, Oxi) that turn green when all thresholds are satisfied and red if any parameter falls outside the acceptable range, in which case measurement is blocked until the issue is resolved. For DCS, pass requires dark counts <2  kHz when lasers are off, signal counts between 10 and 200 kHz when the DCS laser is on, and no cross-talk when the TD-NIRS laser is active (DCS dark counts <3  kHz). For TD-NIRS (here indicated as TRS for simplicity), pass requires per-wavelength IRF/phantom counts within 0.8 to 1.2 Mcps and dark counts below 30 kcps. For the oximeter (here indicated as Oxi for simplicity), pass requires the manufacturer’s built-in quality flag to confirm a valid plethysmographic signal and a stable ${\mathrm{SpO}}_{2}$. These results guide operators in ensuring reliable data collection. Additional details about the software and its protocols have been discussed elsewhere.^89^

Approximately 10% of the subjects were randomly selected to investigate the influence of TD-NIRS and DCS light levels on each other, both in the absence and presence of laser illumination. The background level was measured by integrating the total photon count when no diffuse light was incident on the detector, for both modules, both with and without the counterpart light active. The effect of the counterpart light on the signal was assessed by integrating the photon count with the counterpart light either turned off or on. The influence of a module on the other was then analyzed using the Mann–Whitney test, with a significance level set at p<0.05.

### 2.4 Validation for Clinical Settings

#### 2.4.1 Comparison against commercially available device during a vascular occlusion test

The hDOS device was compared with a commercially available INVOS 5100C (Somanetics, now part of Medtronic, Minnesota, USA) on two different muscles: the *palmaris longus* and *brachioradialis*. Subject demographics (age and biological sex) and the adipose tissue thickness (ATT) were collected prior to the measurements. In particular, the ATT for both *palmaris longus* and *brachioradialis* muscles was measured by means of a portable ultrasound (ECUBEi7, ALPINION MEDICAL SYSTEMS Co., Ltd., Republic of Korea).

The INVOS 5100C and hDOS device were alternately positioned on one muscle or the other of healthy subjects in a randomized order for two subsequent VOTs, with an interval of ∼30  min between each test.

For each measurement, the pertinent VOT-derived parameters, as explained previously, were calculated. To synchronize the two devices, manual markers were utilized that are then used as a reference in postprocessing.

The hDOS TD-NIRS module operates at 1 Hz, whereas the INVOS 5100C provides ${\mathrm{StO}}_{2}$ values every 5 to 6 s at its fastest rate. Therefore, TD-NIRS data were resampled to match the timing of the INVOS 5100C. The responses of the two devices to the VOT, as well as their performance across the two different muscles, were then compared.

The CV was used as a metric to assess intra-muscle variability within the two muscles. The statistical difference between the *palmaris longus* and *brachioradialis* was evaluated using the Wilcoxon signed-rank test, with significance set at p<0.05. In addition, a Bland-Altman plot was employed to visualize and assess whether the ${\mathrm{StO}}_{2}$ measurements from the INVOS 5100C are interchangeable with those of hDOS device.

#### 2.4.2 Characterization of the microcirculation: comparison between healthy subjects and general mixed ICU patients

The clinical study aimed to ensure the device’s efficacy and safety in critical care scenarios, and it was part of the VASCOVID clinical study. Measurements on healthy subjects were conducted at the Institute of Photonic Sciences (ICFO) and were approved by the associated ethical committee (Ref: ICFO_HCP/2012/1).

The clinical protocol carried out at Parc Taulí Hospital received approval from its local Ethics Committee and the Comité d’Investigació amb Medicaments (CEIm) (Ref:2021/3015). The study was conducted in accordance with the Helsinki Declaration of 1975, and revised in 2008.

The purpose of this study is to characterize microvascular reactivity in the *brachioradialis* muscle of patients admitted to the ICU and compare it with that of healthy subjects.

The study includes two different groups: (1) Adult healthy volunteers with no previous history of disease that can affect blood circulation, recruited on a voluntary basis; (2) adult general ICU patients, including septic and nonseptic patients.

Exclusion criteria for the ICU patients included the presence of venous thrombosis in the upper limbs, as well as hematomas or skin lesions on the forearm that could interfere with the placement of the hDOS device’s probe. Hemodynamically unstable patients, defined by uncorrected arterial hypotension or the need for active resuscitation to optimize blood pressure and/or cardiac output, were not included. Participation in the study was voluntary, with informed consent obtained either from the patient or from their legal representative.

Due to the exceptional situation of the pandemic from SARS-CoV-19 infection, if the physical access to the patients’ representatives was not possible, informed consent was obtained verbally, by means of telephone conference, with an external witness, not involved in the study.

Data were collected and managed using REDCap (Research Electronic Data Capture) electronic data capture tools hosted at ICFO.^112^^,^^113^

The vascular reactivity of the subjects was characterized by means of a VOT, as previously described in Section, on the *brachioradialis* muscle, with a pressure applied 50 mmHg above the systolic value. Systolic pressure was measured using a commercial blood pressure monitor on the contralateral arm for the healthy subjects, whereas in the ICU patients it was obtained by invasive blood pressure monitoring. Finally, the differences between the groups were evaluated by means of Mann–Whitney U test with a significance level of p<0.05.

## 3 Results

### 3.1 Precision of the hDOS Device

#### 3.1.1 Variability of key parameters at module level

In a period of time of seven months, a total of 59 IRF measurements and corresponding phantom measurements were collected. The dataset covers 59 days of monitoring and includes over 100 measurement sessions conducted at the hospital.

**Fig. 8:**
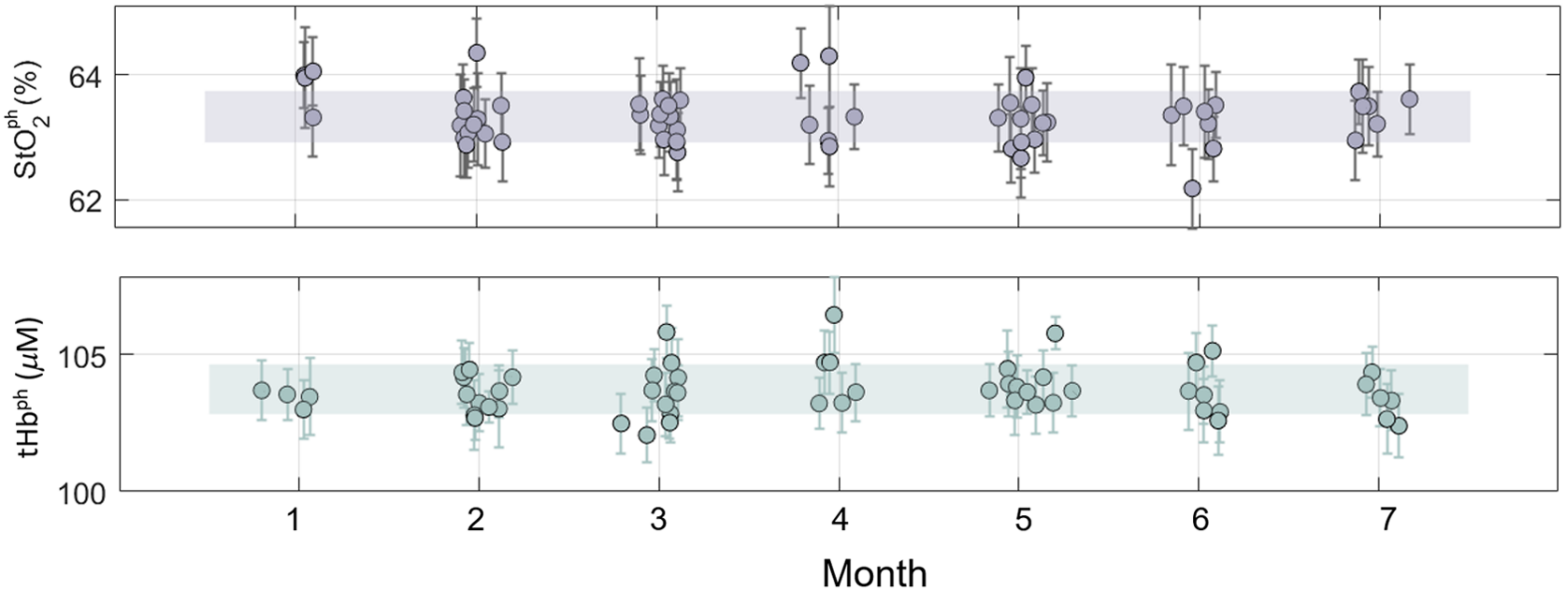
Effective ${\mathrm{StO}}_{2}^{\mathrm{ph}}$ in %(top panel) and ${\mathrm{tHb}}^{\mathrm{ph}}$ in μM (bottom panel) obtained by the solid phantom for 59 days of measurements, grouped by month for clarity. The dots represent the average values, whereas the error bars represent the standard deviation over 20 repetitions. The shaded areas represent the standard deviation over all the measurements.

The CV for the FWHM in the IRF was 2.9% (1.1%) at 685 nm (828 nm), and for the barycenter, the precision was 3.9± 0.02 ns (CV = 0.4%), and at 828 nm it was 3.6±0.02  ns (CV = 0.6%). For $\mu_{a}$, the CV was 0.9% (1.0%) at 685 nm (828 nm). For $\mu_{s}^{\prime }$, the CV was 2.2% (2.3%) at 685 nm (828 nm). When calculating the effective ${\mathrm{tHb}}^{\mathrm{ph}}$, the CV was 1.0%, and for ${\mathrm{StO}}_{2}^{\mathrm{ph}}$, it was 1.2% over the entire measurement period. The intra-phantom variability was 1.4% for ${\mathrm{tHb}}^{\mathrm{ph}}$ and 1.3% for ${\mathrm{StO}}_{2}$. Results for the effective ${\mathrm{tHb}}^{\mathrm{ph}}$ and ${\mathrm{StO}}_{2}^{\mathrm{ph}}$ are shown in Fig. 8 where they have been grouped by month for clarity and a summary of the results is reported in Table 1. Each dot represents the mean, and the error bars represent the standard deviation over 20 consecutive repetitions. The shaded areas represent the standard deviation over the whole period of time considered.

**Table 1:** Coefficient of variation (CV %) calculated across all measurement sessions recorded in seven months for both the full-width-half-maximum (FWHM) of the IRF and the optical properties phantom ($\mu_{a}$ in ${\mathrm{cm}}^{-1}$ and $\mu_{s}^{\prime }$ in ${\mathrm{cm}}^{-1}$). For the barycenter, the standard deviation across all measurement sessions is reported, in ns. A total of 59 IRF and phantom measurements were collected during this period. The $\mu_{a}$ in ${\mathrm{cm}}^{-1}$ was employed to calculate the effective ${\mathrm{tHb}}^{\mathrm{ph}}$ and ${\mathrm{StO}}_{2}^{\mathrm{ph}}$.

	IRF		Phantom			
	FWHM (%)	Barycenter (ns)	$\mu_{a}$ (%)	$\mu_{s}^{\prime }$ (%)	${\mathrm{tHb}}^{\mathrm{ph}}$ (%)	${\mathrm{StO}}_{2}^{\mathrm{ph}}$ (%)
685 nm	2.9	0.02	0.9	2.2	1.0	1.2
828 nm	1.1	0.02	1.0	2.3		

When fitting each phantom with the first phantom of the month (e.g., all phantom measurements acquired in month 3 are fitted with the first IRF acquired in month 3), we obtained a higher, but not statistically significantly different, variability in the retrieval of $\mu_{a}$ (CV = 2.8% for both 685 and 828 nm; p=0.52) and $\mu_{s}^{\prime }$ (CV = 2.5% for both 685 and 828 nm; p=0.75). This higher variability translated to a CV = 2.8% in the effective ${\mathrm{tHb}}^{\mathrm{ph}}$ and a CV = 1.0% in the ${\mathrm{StO}}_{2}^{\mathrm{ph}}$. More details about the variability on the optical properties of the phantom are shown in Supplementary Material.

**Fig. 9:**
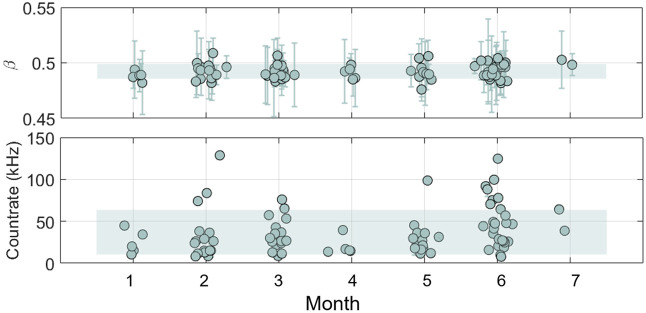
Top panel: β parameter obtained for 7 months worth of measurement. The dots represent the average values over 60 s of baseline, with averaging time of 10 s, whereas the error bars represent the standard deviation. Botton panel: count rate for DCS in kHz for each subject admitted in the clinical study. Dots, each representing a single subject, correspond to the mean values, whereas error bars the standard deviation over 20 s of baseline measurement. The green shaded area correspons to the standard deviation over all the measurements.

The photon count rate and β parameter were analyzed for N=137 subjects and shown in Fig. 9 (each represented by a single point with its mean and standard deviation calculated over the baseline). The photon count rate was subject-dependent and showed a weak positive trend (Spearman’s correlation, ρ=0.3, p=0.03). As *in vivo* conditions vary across subjects, the recruitement of subjects with better signal might have contributed to this weak positive trend. The β parameter had an average value of β=0.49±0.01 with a CV = 1.9%, recorded in seven months worth of measurements. No significant trends were observed for β over time (Spearman’s correlation, ρ=0.2, p=0.06). This result suggests that there is no degradation in the system over the time of measurements at the hospital.

Further details about DCS quality parameters are available in the Supplementary Material.

#### 3.1.2 Test-retest variability

**Fig. 10:**
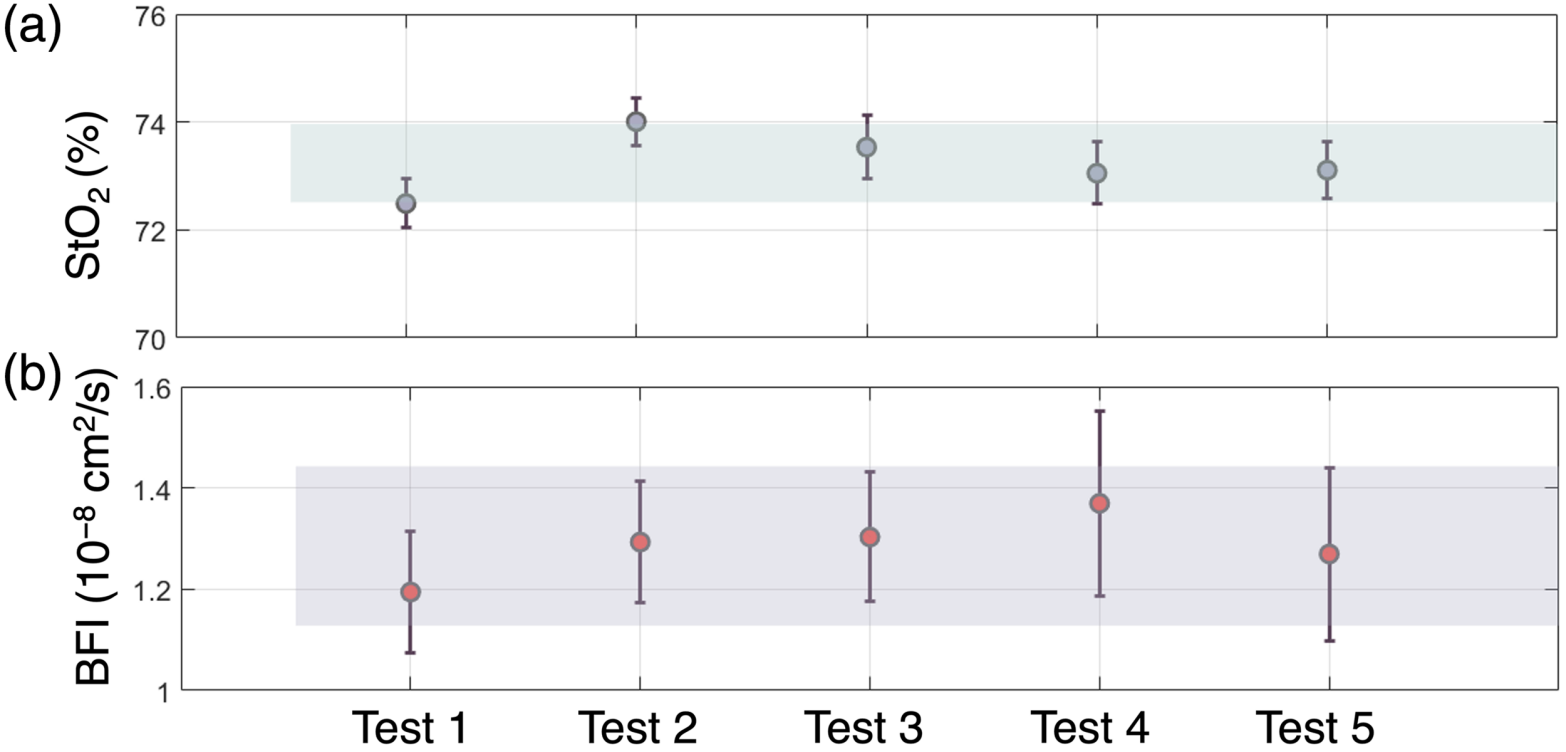
(a) ${\mathrm{StO}}_{2}$ values obtained over five repositioning. The dots represent the mean values, whereas the error bars are the standard deviations calculated over 200 acquisitions. The shaded area represent the standard deviation, centered around the mean over the five tests.

As a test-retest evaluation, the hDOS device’s probe was replaced five times onto the *brachioradialis* muscle of a healthy female subject (32 years old). The results are summarized in Fig. 10, where the mean values of ${\mathrm{StO}}_{2}$ and BFI across the five tests are shown. Over the entire experiment, the inter-repetition CV was 1.2% and 12.6% for ${\mathrm{StO}}_{2}$ and for BFI, respectively. The within-test variability was found to be CV<0.8% for ${\mathrm{StO}}_{2}$ and CV<15.2% for BFI and it was similar to what was found in Ref. 111, although ${\mathrm{StO}}_{2}$ was better with hDOS (1.2% reported here compared with 8.5% in Ref. 111).

#### 3.1.3 Dependence of precision with respect to absolute values

**Fig. 11:**
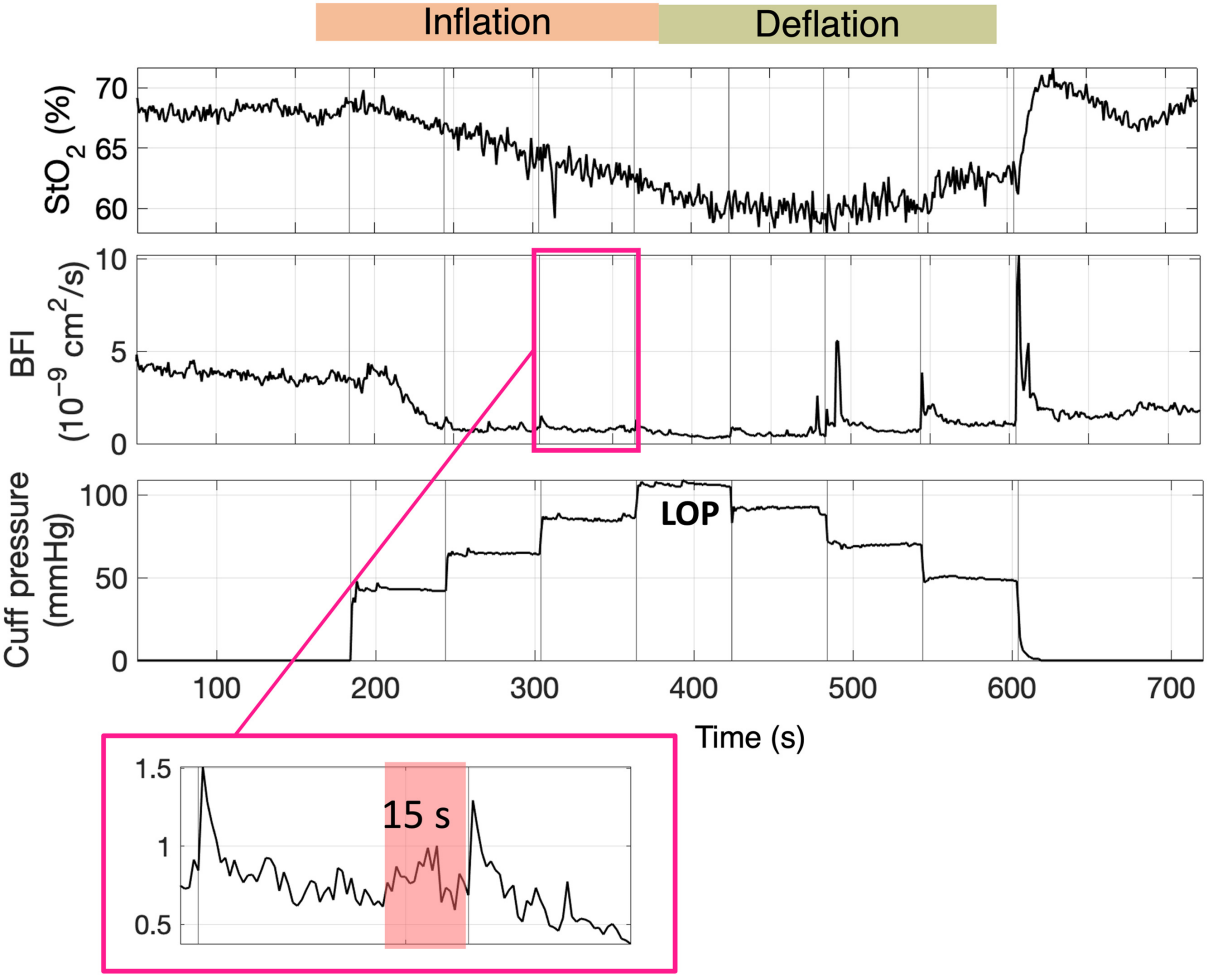
Example of VOT-LOP protocol for tissue oxygen saturation [${\mathrm{StO}}_{2}$ in % and blood flow index (BFI) in ${\mathrm{cm}}^{2}/s$]. The cuff was inflated and deflated in step of 40%, 60%, 80%, and 100% of the LOP, with occluding pressure maintained for 1 min each time. Mean value, standard deviation, and CV are calculated in the last 15 s of occlusion for each interval.

**Fig. 12:**
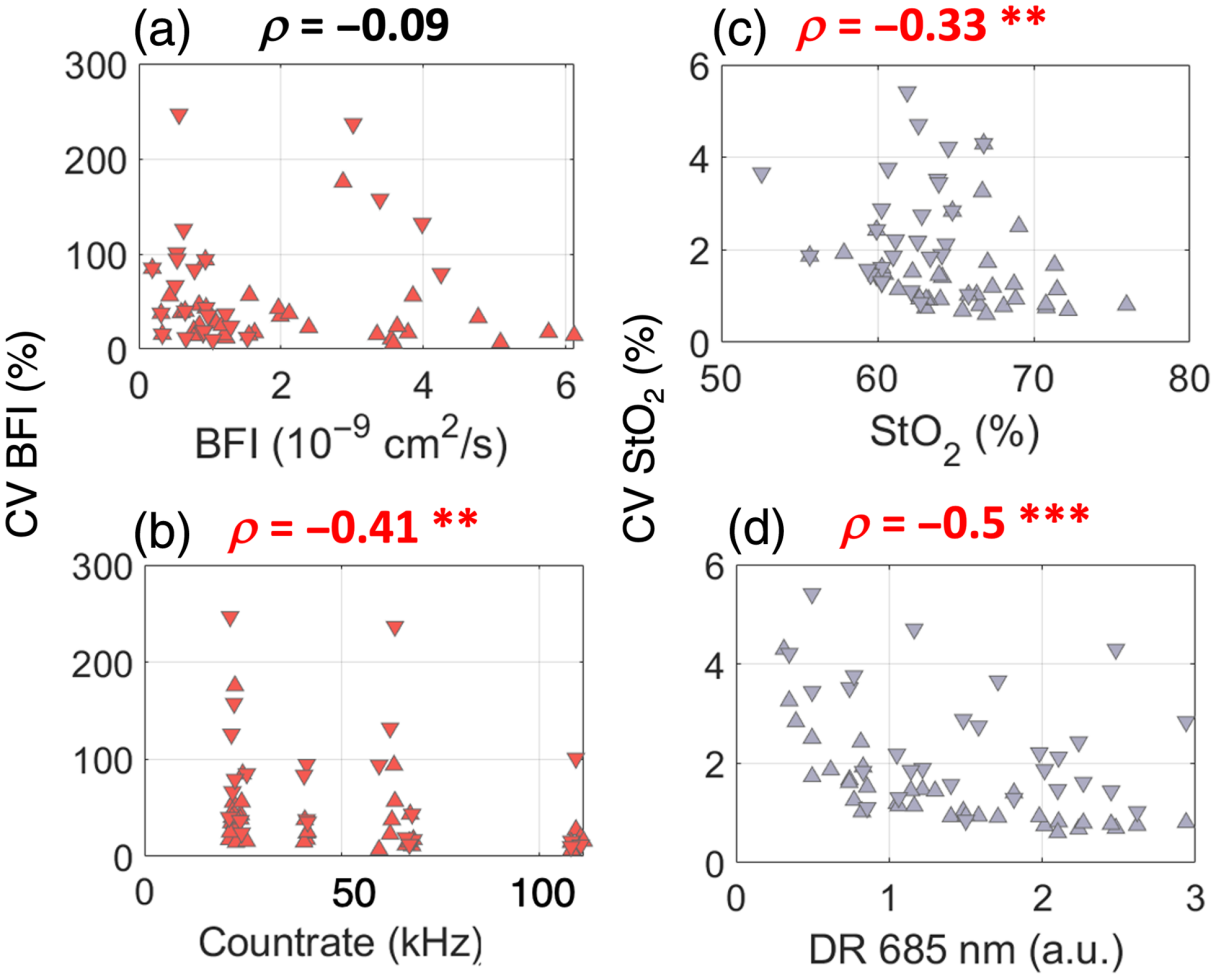
Coefficient of variation (CV in %) of BFI with respect to (a) the mean values of BFI (in ${\mathrm{cm}}^{2}/s$) and (b) to the count rate (in kHz), retrieved during the inflation/deflation periods; CV of tissue oxygen saturation (${\mathrm{StO}}_{2}$) with respect to (c) the mean value of ${\mathrm{StO}}_{2}$ (in %) and (d) the dynamic range (DR) of the wavelength 685 nm.

Seven (N=7) healthy subjects were enrolled to evaluate the variability at different light levels of both ${\mathrm{StO}}_{2}$ and BFI. In Fig. 11, an example is reported for a single subject. As displayed in Fig. 12, it can be observed that for both BFI and ${\mathrm{StO}}_{2}$, the CV was dependent on the mean value retrieved during the various periods of inflation/deflation, with higher CV observed during deflation for both ${\mathrm{StO}}_{2}$ and BFI (panels a and c, respectively). The CV BFI exhibited similar behavior, showing smaller variability around higher count-rates (panel b). As expected, CV ${\mathrm{StO}}_{2}$ decreased with higher dynamic ranges of the acquired TD-NIRS curves (panel d). A statistically significant correlation was obtained between the CV BFI and count rate (ρ=−0.41, p=0.001). No statistically significant correlation was found between count rate and the increase in BFI (ρ=−0.09, p=0.46), nor between CV BFI and BFI (ρ=−0.19, p=0.15). For ${\mathrm{StO}}_{2}$, a statistically significant correlation was found between CV ${\mathrm{StO}}_{2}$ and the dynamic range (DR) at 685 nm (ρ=−0.5, p<0.001), as well as with ${\mathrm{StO}}_{2}$ (ρ=−0.33, p=0.007). Finally, no statistically significant correlation was found when comparing ${\mathrm{StO}}_{2}$ and the dynamic range at 685 nm (ρ=0.08, p=0.5).

#### 3.1.4 Optical interference

**Fig. 13:**
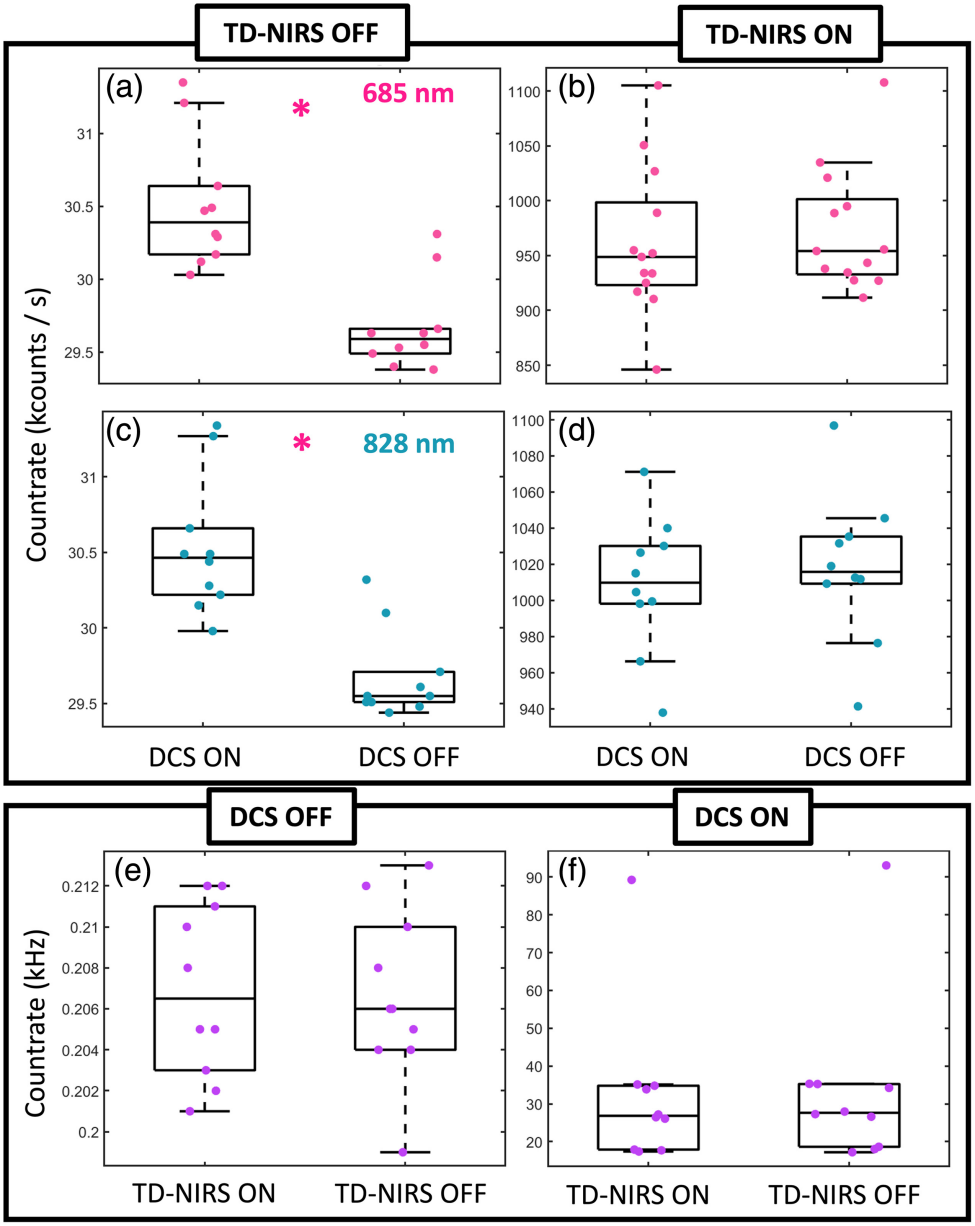
Boxplots illustrating the influence of the DCS signal on the TD-NIRS total background count rate (TD-NIRS OFF) when the DCS was ON or OFF for wavelength 685 (a) and 828 (c) nm; (b) influence of the DCS signal on the total count rate of the TD-NIRS module (TD-NIRS ON) when the DCS was ON or OFF for the wavelengths 685 (b) and 828 (d). Influence of the TD-NIRS signal when the TD-NIRS lasers are either ON or OFF, when the DCS lasers was either OFF (e) or ON (f). A statistically significant difference is depicted by “*” when p<0.05.

Results for the assessment of the presence of optical interference, is reported in Fig. 13.

**Fig. 14:**
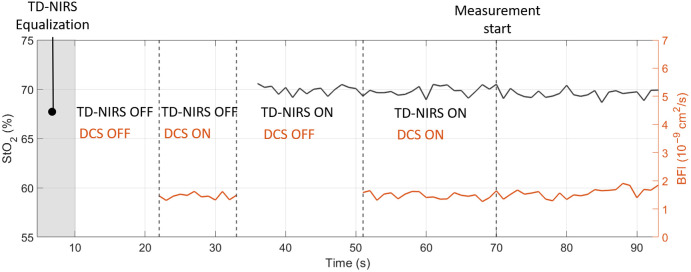
Example of ${\mathrm{StO}}_{2}$ (in black solid line) and BFI (in orange solid line) time traces during the quality assessment phase. Dashed vertical llines denote the changes in ON/OFF cycles. A grey shaded area represents the equalization phase for TD-NIRS to reach the desired count rate $10^{6}\;\text{counts}/s$.

The background count rate for the TD-NIRS was significantly higher when the DCS laser was emitting at full power (p<0.001 for both 685 and 828 nm). On the other hand, the background count rate of the DCS is not affected by the TD-NIRS lasers shining (p=0.48). Finally, a difference in the count rate when the TD-NIRS lasers are emitting and the DCS was either ON or OFF has not been found (p=0.15 for 685 nm and p=0.60 for 828 nm). Similar results are obtained when the DCS laser is emitting, and the TD-NIRS lasers are switched ON and OFF (p=0.31). The results suggest that the filters are functioning as expected, effectively preventing significant interference. The increase in background count rate when the DCS was ON, though statistically significant, was minimal and spread across the entire temporal window. Despite this increase, the system’s performance remains uncompromised, as sufficient dynamic range was maintained to reliably fit the DTOF and extract robust optical properties from the signal. A representative result is shown in Fig. 14 where the ${\mathrm{StO}}_{2}$ and the BFI of a single patient is reported during the automatic QP. In this particular case, the CV for the BFI was 7.5% when the DCS laser was ON, whereas the TD-NIRS lasers were OFF. Conversely, when the TD-NIRS module was ON and the DCS was OFF, the CV for the $\mu_{a}$ and $\mu_{s}^{\prime }$ was less than 1% at both wavelengths, with a resulting CV for ${\mathrm{StO}}_{2}$ at 0.6% and for tHb at 0.5%. When both the TD-NIRS and DCS modules were ON, the CV for BFI remained at 7.5%, whereas the CV for ${\mathrm{StO}}_{2}$ and tHb increased slightly to 0.8%, and the CV for $\mu_{a}$ and $\mu_{s}^{\prime }$ was less than 1.2% at both wavelengths.

### 3.2 Validation for Clinical Settings

#### 3.2.1 Comparison against a commercially available device

**Fig. 15:**
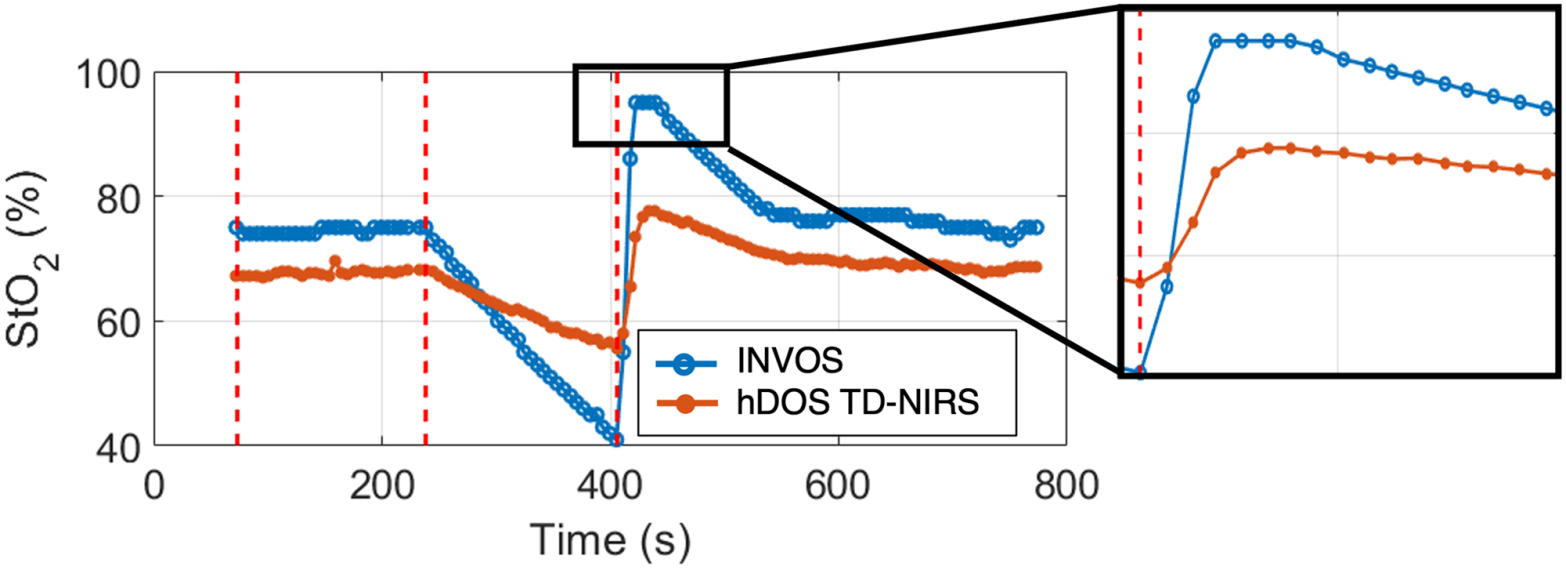
Example of the time trace of a simultaneous measurement of hDOS device (orange line resampled at the INVOS 5100C sampling time) and INVOS 5100C (blue line). The dashed lines represent the start of the measurement, after the data quality assessment phase, inflation and deflation time. The ${\mathrm{StO}}_{2}$ hyperemic peak is highlighted in the zoomed window.

A total of ten subjects (six females) with a mean age of 28±5 years old and a BMI of $24.9\pm 2.3\;\mathrm{kg}/m^{2}$ were included in this study. In addition, the ATT over the two measured muscles was 4.0±0.1  mm for the *brachioradialis* and 4.4±0.1  mm for the *palmaris longus* which were not statistically significantly different from each other (p=0.67). An example of hDOS device and INVOS 5100C time traces is reported in Fig. 15. The response to the ischemic challenge was lower in the hDOS device. Moreover, the ${\mathrm{StO}}_{2}$ in the INVOS 5100C was limited to a maximum value of 95% and a minimum value of 15%, which makes the extraction of the AUC unreliable (see the zoomed area in Fig. 15). A summary of the VOT-derived parameters, their medians, and interquartile ranges is shown in Table 2 for both devices and muscles.

**Table 2:** Comparison of hDOS TD-NIRS and INVOS 5100C values obtained for the *brachioradialis* and *palmaris longus* during the VOT.

	hDOS TD-NIRS		INVOS 5100C	
	Brachioradialis	Palmaris longus	Brachioradialis	Palmaris longus
${\mathrm{StO}}_{2}$ (%)	72.0 [69.9 to 72.5]^*^	70.7 [68.4 to 73.0]	76.4 [72.8 to 79.4]	74.0 [69.0 to 81.7]
${\mathrm{DeO}}_{2}$ (%/min)	−4.6 [−5.2 to −3.7]^*^^,^^**^	−5.4 [−6.6 to −4.4]^*^	−10.9 [−14.4 to −8.9]	−11.4 [−13.6 to −10.3]
${\mathrm{ReO}}_{2}$ (%/s)	0.9 [0.7 to 1.2]^*^^,^^**^	1.2 [1.0 to 1.6]^*^	2.7 [2.2 to 3.7]	2.8 [2.3 to 3.1]
AUC ${\mathrm{StO}}_{2}$ (%· min)	6.4 [3.6 to 8.5]	7.4 [5.6 to 7.7]	7.5 [4.9 to 8.5]	7.4 [6.6 to 8.8]

Values are median and first and third interquartile range values in square brackets.

* Represents statistically significant difference between the hDOS TD-NIRS and INVOS 5100C. In both cases, the significance was set for p<0.05.

** Represents statistically significant difference between *brachioradialis* and *palmaris longus.*

The hDOS TD-NIRS ${\mathrm{StO}}_{2}$ presents a smaller interquartile range, particularly in ${\mathrm{DeO}}_{2}$ and ${\mathrm{ReO}}_{2}$. Concerning intersubject variability, a CV of 3.3 (5.0)% for hDOS device in the *brachioradialis* (*palmaris longus*) was obtained, compared with 6.1 (9.6)% for INVOS 5100C. In addition, when intrasubject variability was considered, an average of 0.4 (0.6)% was found for hDOS device versus 0.8 (1.0)% for the *brachioradialis* (*palmaris longus*) in INVOS 5100C, respectively. Statistical differences in the positioning of the probe were not observed for any of the variables retrieved by INVOS 5100C. However, differences were found in the ${\mathrm{DeO}}_{2}$ (p=0.004) and ${\mathrm{ReO}}_{2}$ (p=0.009) variables between the *brachioradialis* and *palmaris longus* when examining TD-NIRS-hDOS. In the *brachioradialis*, differences were noted in the ${\mathrm{StO}}_{2}$ baseline (p=0.02), as well as in ${\mathrm{DeO}}_{2}$ (p<0.001) and ${\mathrm{ReO}}_{2}$ (p<0.001) when comparing TD-NIRS and INVOS 5100C. No significant differences were found in the AUC ${\mathrm{StO}}_{2}$ (p=0.61). In the *palmaris longus*, differences were observed only in ${\mathrm{DeO}}_{2}$ and ${\mathrm{ReO}}_{2}$ (p<0.001).

**Fig. 16:**
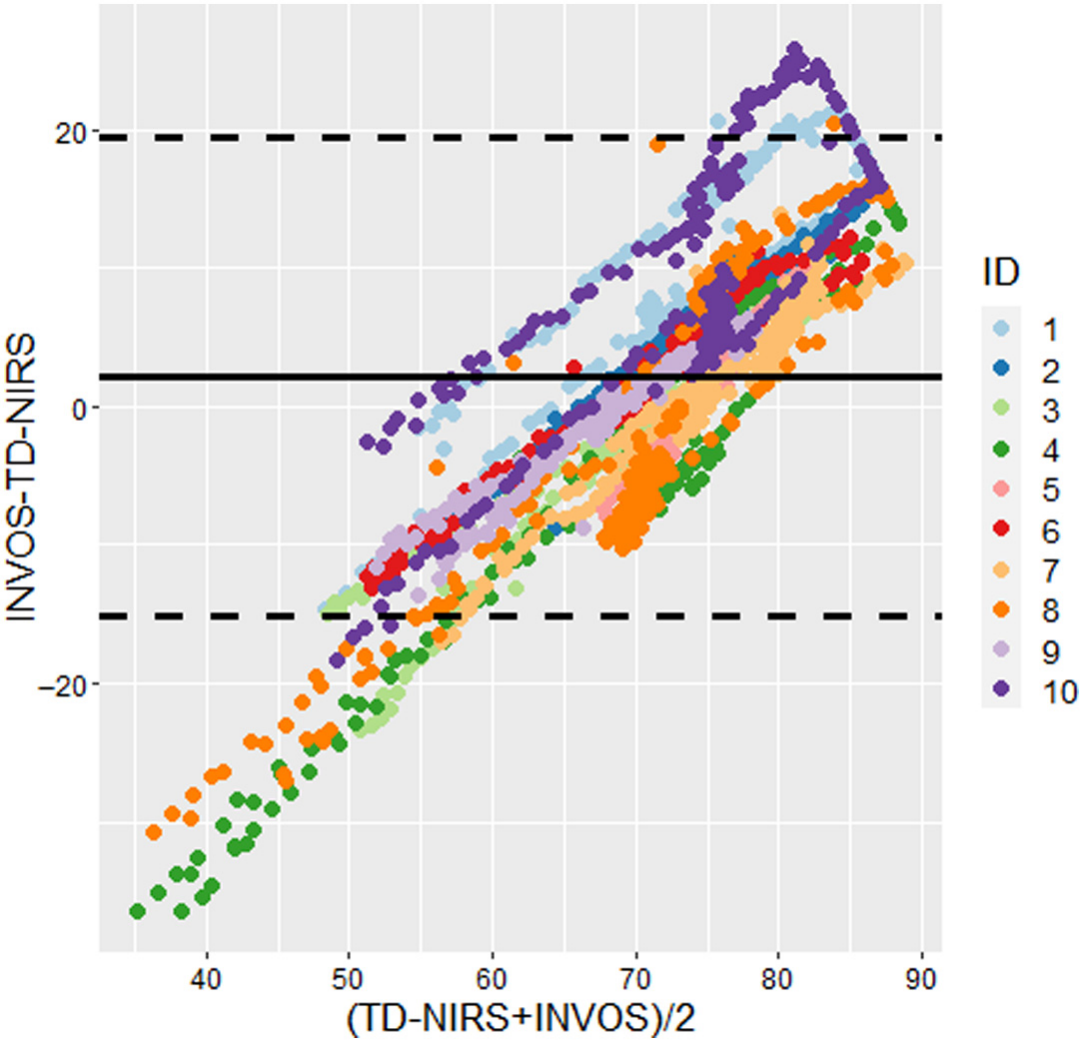
Bland-Altman plot for ${\mathrm{StO}}_{2}$ as measured by INVOS 5100C (indicated as INVOS) and hDOS TD-NIRS (indicated as TD-NIRS). Colors represent all the subjects ID included in the study (N=10). Both positions of measurement (*brachioradialis* and *palmaris longus*) are taken into consideration in the plot.

In Fig. 16 a Bland-Altman plot is reported where for each subject (in different colors), the differences between INVOS 5100C and hDOS ${\mathrm{StO}}_{2}$ are plotted against their average values at each time point of the VOT. The black solid line correspond to the bias, whereas the black dashed lines correspond to ±1.96 times the standard deviation. A bias of +2.14% is reported, which is not representative in this case as there is a nonzero slope (R=0.72, p<0.001), confirming that the two devices differ from each other in a nontrivial manner. Furthermore, this difference is not subject dependent. In fact, all subjects display a significant nonzero slope.

#### 3.2.2 Characterization of the microcirculation: comparison between healthy subjects and general mixed ICU patients

Thirty-seven (N=37) healthy subjects and one hundred (N=100) general ICU patients were recruited. The demographic, clinical, and morphological data are summarized in Table 3, whereas the optical data, as well as baseline and median responses to the ischemic challenge, are presented in Table 4.

**Table 3:** Demographic data. Median and interquartile range (25% and 75%) is reported in square brackets.

	General mixed ICU N=100	Healthy N=37
Gender (F %)	40	60
Age (years old)	66 [56 to 73]^*^	28 [26 to 34]
BMI (${\mathrm{kg}/m}^{2}$)	28.4 [25.0 - 32.8]^*^	23.9 [22.0 to 25.0]
ATT (mm)	3.5 [2.6 to 6.4]	4.0 [3.4 to 5.5]
Circumference (cm)	28.0 [25.2 to 30.0]^*^	24.5 [23.2 to 26.0]
Hemoglobin (g/dL)	10.1 [8.6 to 11.5]	N/A
MAP (mmHg)	80.0 [75.0 to 88.0]^*^	86.8 [82.0 to 93.0]
${\mathrm{SpO}}_{2}$ (%)	97.0 [95.8 to 99.0]^*^	99.0 [97.7 to 99.0]
HR (beats/minute)	78.0 [68.0 to 90.0]^*^	63.7 [58.3 to 74.1]

BMI, body mass index; ATT, adipose tissue thickness; MAP, mean arterial pressure; ${\mathrm{SpO}}_{2}$, peripheral tissue oxygenation; HR, heart rate; N/A, information not available or not applicable.

* Indicates difference between the general mixed ICU and healthy population with a significance level p<0.05 according to Wilcoxon rank-sum test.

**Table 4:** Optical data obtained for the three subjects’ cohorts. The mean values and standard deviations (mean ± std. dev) of tHb, ${\mathrm{StO}}_{2}$, BFI, OEF, and ${\mathrm{MMRO}}_{2}$ are calculated over 30 s prior to the occlusion interval. Median and interquartile range (25% and 75%) of ${\mathrm{DeO}}_{2}$, ${\mathrm{ReO}}_{2}$, AUC ${\mathrm{StO}}_{2}$, and AUC BFI are reported in square brackets.

	General Mixed ICU N=100	Healthy N=37
$\mu_{a}$ 685 (${\mathrm{cm}}^{-1}$)	0.22 ± 0.07	0.22 ± 0.06
$\mu_{a}$ 828 (${\mathrm{cm}}^{-1}$)	0.21 ± 0.05	0.23 ± 0.06
$\mu_{s}^{\prime }$ 685 (${\mathrm{cm}}^{-1}$)	9.25 ± 1.11^*^	8.62 ± 0.77
$\mu_{s}^{\prime }$ 828 (${\mathrm{cm}}^{-1}$)	8.65 ± 1.22^*^	8.18 ± 0.85
tHb (μM)	104.04 ± 26.75	112.88 ± 27.82
${\mathrm{StO}}_{2}$ (%)	67.05 ± 5.54^*^	70.33 ± 3.73
BFI ($10^{-8}\;{\mathrm{cm}}^{2}/s$)	0.49 ± 0.50^*^	0.53 ± 0.31
OEF (%)	30 ± 6^*^	28 ± 4
${\mathrm{MMRO}}_{2}$ ($10^{-8}\%\cdot {\mathrm{cm}}^{2}/s$)	1.39 ± 1.23^*^	2.09 ± 1.1
${\mathrm{DeO}}_{2}$ (%/min)	−4.3 [−5.7 to −3.3]	−4.1 [−5.2 to −3.1]
${\mathrm{ReO}}_{2}$ (%/s)	0.8 [0.5 to 1.1] ^*^	1.1 [0.9 to 1.6]
AUC ${\mathrm{StO}}_{2}$ (%min)	3.8 [2.0 to 6.5] ^*^	7.7 [5.2 to 9.3]
AUC BFI ($10^{-6}\;{\mathrm{cm}}^{2}$)	2.2 [1.1 to 3.3] ^*^	4.0 [2.6 to 5.7]

* Indicates difference between the general mixed ICU and healthy population with a significance level p<0.05 according to Wilcoxon rank-sum test.

Statistically significant differences were observed between the ICU and healthy groups in age, BMI, arm circumference, ${\mathrm{SpO}}_{2}$, and baseline HR (p<0.001). A significant difference was also found in MAP between the two groups (p=0.02).

Significant differences were found in $\mu_{s}^{\prime }$ at both wavelengths (p<0.001). Healthy group showed a significantly higher ${\mathrm{StO}}_{2}$ with respect to the general mixed ICU group (p<0.001). A significant difference in BFI was detected (p=0.03) with higher values in the healthy group. ${\mathrm{MMRO}}_{2}$ was found to be significantly reduced in the ICU population (p<0.001). During the VOT, significantly lower values were identified for the ${\mathrm{ReO}}_{2}$, AUC ${\mathrm{StO}}_{2}$ and AUC BFI in the ICU population compared with healthy subjects (all p<0.001).

## 4 Discussion

In this paper, the hDOS device is introduced as a versatile, multimodal system that integrates advanced TD-NIRS and DCS technologies, along with essential accessories such as a pulse oximeter and an automated tourniquet for VOTs and baseline metabolism assessment.

The validation of this platform went beyond basic performance metrics, emphasizing its robustness and suitability for independent clinical use. Although optimized for intensive care, its adaptability extends to various settings, including laboratory environments, clinics, and more demanding conditions such as the operating room and emergency care unit.

The device underwent rigorous testing, with over 200 h of use across ∼150 measurement sessions.

A user-friendly software improves usability with an intuitive interface, quality feedback, and real-time safety checks to ensure compliance with safety standards. The multimodal probe, equipped with a force sensor, touch sensor, and accelerometer, aids in standardizing probe pressure, improving measurement quality, and enabling data rejection when necessary. The accelerometer detects motion artifacts, whereas the capacitive touch sensor ensures continuous tissue contact for accurate measurements.^92^^,^^114^^,^^115^

Deployed in the ICU for seven months, the device underwent evaluations, including tests on tissue-mimicking phantoms and *in vivo* assessments to gauge its stability, reproducibility, and performance in a clinical setting. As of now, the device has been in use for 24 months, with over 500 sessions conducted on 410 patients across 12 clinical protocols. The quality and stability of the device continue to be monitored and will be reported alongside the results of each specific clinical study. To note that for some of these assessments, a replica device has been utilized. These replicas differ only in having slightly improved and more robust electronics, but are optically identical. Specifically, it has been the focus of the work of Ref. 15 where a comprehensive comparison involving ten TD-NIRS devices based on the same hDOS TD-NIRS technology was presented. They in fact revealed a remarkably high level of reproducibility and accuracy in the retrieval of optical parameters from well-characterized tissue-mimicking phantoms among different replicas, which is promising for the consistency and reliability of TD-NIRS devices manufactured at scale and utilized in this very same platform. In particular, for a similar system, in Ref. 116, authors report a variability better than 1.2% on different types of phantoms and different, bulkier implementations of TD-NIRS technology, with comparable optical properties to those employed with hDOS device.

For hDOS TD-NIRS, a variability in ${\mathrm{tHb}}^{\mathrm{ph}}$ and ${\mathrm{StO}}_{2}^{\mathrm{ph}}$ was found to be below 1.2%. The focus was on assessing a day-to-day variability, not on validating absolute values. These results are consistent with findings from Ref. 111, where variability was 3.0% over nearly 10 months. Monthly calibration with the IRF showed less than 3% variability in effective ${\mathrm{tHb}}^{\mathrm{ph}}$ and ${\mathrm{StO}}_{2}^{\mathrm{ph}}$, despite minor, statistically insignificant variations in optical properties ($\mu_{a}$ and $\mu_{s}^{\prime }$).

DCS stability, well-documented in Refs. 21, 23, 24, 91, 94, and 117–119, was confirmed by monitoring count rate and β parameters in *in vivo* measurements. No significant trends were observed, indicating stable performance during data acquisition.

The precision of the hDOS device was evaluated through test-retest and precision analysis concerning absolute values. In a test-retest study on a single subject, TD-NIRS results were consistent with literature for both TD-NIRS^14^^,^^120^ and DCS.^90^^,^^121^ The CV for ${\mathrm{StO}}_{2}$ with hDOS was notably lower at 1.2% compared with 8.5% reported previously^111^ and also superior to MOXY (Fortiori Design LLC, Minnesota, US) and Portamon (PortaMon, Artinis, Medical System, The Netherlands) devices, which reported CVs of less than 2.5%.^122^ The hDOS TD-NIRS shows a better precision with respect to a novel commercially available wearable device (Train.Red FYER) where a CV of 5% was reported. Although the probe integrates a force sensor to monitor applied pressure, the effect of probe pressure on test—retest variability was not investigated in this study. This is an important factor that we plan to evaluate in future dedicated studies. Regarding the evaluation of the precision with respect to absolute values, CV was found to be dependent on light levels for both ${\mathrm{StO}}_{2}$ and BFI. During dynamic phases, such as deoxygenation and reoxygenation, a lower ${\mathrm{StO}}_{2}$ resulted in a reduced SNR and increased CV. Future work will aim to improve precision across all measurement ranges using real-time optimization algorithms and fast tunable filters. Despite this, the precision was better than 4% across the ${\mathrm{StO}}_{2}$ range. BFI variability increased during full arterial occlusion, but this does not impact measurements as BFI during occlusion only confirms ischemia. Evaluating *in vivo* variability, especially in skeletal muscle, remains complex and underexplored compared with brain studies.

Precision studies in tissue mimicking phantoms and *in vivo* provide a valuable benchmark when translating the usefulness of these technologies to clinical applications. On the other hand, it is also critical to assess how it compares to a less expensive commercially available device, such as the INVOS 5100C. The INVOS 5100C, such as the hDOS TD-NIRS system, uses near-infrared light for tissue oxygenation assessment but relies on the so-called SRS algorithm/probe.^1^^,^^2^ Although valued for its cost-effectiveness and ease of use, it only measures tissue oxygenation without providing direct indicators of perfusion and metabolism. Variability in findings from NIRS-VOT studies^123^ underscores the challenge of uniform standards, an issue addressed in the *in vivo* validation of the VASCOVID project. The key difference between these technologies lies in their assumptions: the INVOS 5100C assumes light attenuation is primarily due to absorption and that scattering depends linearly on wavelength,^124^^,^^125^ deriving a tissue saturation index. In contrast, the TD-NIRS system in the hDOS device calculates absolute absorption values without such assumptions, overcoming limitations of light penetration depth by adjusting injected power within safety limits. Literature consensus supports that TD-NIRS offers superior accuracy, precision, and repeatability compared with CW-NIRS methods.^99^^,^^126^ This suggests that clinical systems such as the INVOS 5100C may provide less accurate data, especially during VOTs. Although definitive *in vivo* comparisons are challenging due to varying algorithms and lack of gold standards, hDOS TD-NIRS demonstrated greater consistency with lower intersubject and intrasubject variability when comparing baseline ${\mathrm{StO}}_{2}$ to INVOS 5100C. Furthermore, as shown in the representative example reported in the Supplementary Material, $\mu_{s}^{\prime }$ remained stable at both 685 nm and 828 nm during the protocol, yet the wavelength-dependent differences between the two traces during the VOT may still influence oxygenation estimates. A previous simulation study^125^ has demonstrated that even modest, unaccounted scattering changes can bias CW-NIRS ${\mathrm{StO}}_{2}$ by up to 10%, underscoring the advantage of TD-NIRS in separating absorption and scattering contributions. Statistical differences were observed in VOT-derived parameters when comparing the *brachioradialis* and *palmaris longus* positions for INVOS 5100C but not for hDOS TD-NIRS. hDOS TD-NIRS showed distinctions in ${\mathrm{DeO}}_{2}$ and ${\mathrm{ReO}}_{2}$ between probe positions. Specifically, in the *brachioradialis*, differences in ${\mathrm{StO}}_{2}$ baseline, ${\mathrm{DeO}}_{2}$, and ${\mathrm{ReO}}_{2}$ were noted, whereas in the *palmaris longus*, differences were seen in ${\mathrm{DeO}}_{2}$ and ${\mathrm{ReO}}_{2}$. A Bland-Altman plot analysis revealed a 2.1% which was not representative due to a nonzero slope. This highlights that the two devices cannot be used interchangeably and bias cannot be corrected by using a simple correction factor. Controlled phantom experiments could provide an important means of systematically investigating discrepancies between TD-NIRS and CW-NIRS measurements. In this study, we have instead focused on in vivo comparisons during VOTs, consistent with prior work in this field. Independent validation or comparison of TD-NIRS is beyond the scope of this work, but the interested reader can refer to the literature where similar TD-NIRS systems were directly compared with clinical CW-NIRS.^29^^,^^127^^,^^128^

Finally, a total of 37 healthy young subjects and 100 general mixed ICU patients were recruited for the clinical study where a 3 min VOT was performed. Literature suggests that fixed time thresholds could introduce variability in ${\mathrm{ReO}}_{2}$ changes.^129^ On the other hand, due to the slower deoxygenation rate observed with hDOS, achieving a consistent 40% ${\mathrm{StO}}_{2}$, as suggested,^129^ threshold upon cuff release was challenging. This device offers a precise automatized VOT protocol thereby reducing any additional variability due to operator.

Optical and hemodynamic properties reported are similar to those in Ref. 120 for the *brachioradialis* muscle. The hDOS device successfully differentiated healthy microcirculation from impaired states in the general mixed ICU group, particularly in the baseline ${\mathrm{StO}}_{2}$, BFI and ${\mathrm{MMRO}}_{2}$. Also, VOT-derived parameters showed differences in parameters related to microvascular reactivity such as ${\mathrm{ReO}}_{2}$, AUC ${\mathrm{StO}}_{2}$ and AUC BFI.

These findings align with literature,^32^^–^^34^^,^^34^^–^^48^^,^^130^^–^^132^ although studies often use the thenar muscle due to its accessibility.

It should be noted that demographic differences (age, sex, and BMI) existed between the ICU and the healthy control groups, which may have influenced the observed hemodynamic responses. Although matching across these variables would be ideal, recruiting such a control population is not straightforward and is not the core purpose of this study. In the present study, the focus was placed on describing the device performance and reporting whether group-level differences could be detected as a side finding. More advanced analyses, including mixed-effects modeling to account for demographic and physiological covariates, are ongoing and will be presented as part of the full clinical study.

The lack of standardized VOT protocols for NIRS technology is another significant concern. Variations in probe positioning, cuff size, inflation pressures, and VOT durations across studies complicate comparisons.^31^^,^^33^^,^^46^^,^^52^^,^^56^^,^^60^^,^^65^^,^^74^^,^^111^^,^^129^^,^^133^^–^^141^

On the other hand, when comparing our results specifically for the healthy population with what is present in the literature, a high variability is shown in the reported baseline values for ${\mathrm{StO}}_{2}$ in healthy subjects. In fact, baseline varies across devices generally ranging between 65% to 87%. Also ${\mathrm{DeO}}_{2}$, ${\mathrm{ReO}}_{2}$, and AUC ${\mathrm{StO}}_{2}$ differ widely in the reported healthy populations, which reflects both methodological differences (e.g., how the fitting point are chosen) and device sensitivities (e.g., source and detector distance in the probe, technology used, etc.) The hDOS TD-NIRS presented in this work for the healthy group, it shows slower ${\mathrm{DeO}}_{2}$ and ${\mathrm{ReO}}_{2}$ of what is normally reported. For example, ${\mathrm{ReO}}_{2}$ reported in the literature, ranges from ≈1.2 to ≈9.5%/s. A comparison with AUC ${\mathrm{StO}}_{2}$ is more complex due to inconsistent reporting. In particular, the variability on ${\mathrm{ReO}}_{2}$ and AUC ${\mathrm{StO}}_{2}$ are due also to variations in protocols where VOT of 3 min or 5 min duration are often used.^33^^,^^47^^,^^53^^,^^76^^,^^80^^,^^129^^,^^133^^,^^142^^,^^143^ These considerations stress the need of a standardized way of measuring the microcirculation in the healthy and ICU population.

The hDOS device demonstrates promising clinical applications. In critical care, it aids in assessing tissue perfusion and oxygenation, monitoring microcirculatory changes, and evaluating endothelial dysfunction, among others.

## 5 Conclusion

The hDOS device is a hybrid diffuse optical platform that has been developed for application in critical care, but given its performances it can find potential applications in many other fields, such as operating rooms and emergency care. The platform has been proven to be more accurate than a commercially available device.

## Supplementary Material

10.1117/1.JBO.30.11.115004.s01

## Data Availability

The data that support the findings of this study will be openly available on CORA, Repositori de dades de Recerca repository at https://doi.org/10.34810/data2733.
