# Supplementary material for: “hDOS”: an automated hybrid diffuse optical device for real-time noninvasive tissue monitoring: precision and in vivo validation
Source: J Biomed Opt. 2025 Nov 18;30(11):115004. doi: 10.1117/1.JBO.30.11.115004 (PMC12626046; doi:10.1117/1.JBO.30.11.115004)
Supplement: Supplementary file 1 [file JBO_030_115004_SD001.pdf]

Supplementary Material: “hDOS”: An automated hybrid diffuse optical device for real-time non-invasive tissue monitoring—precision and *in vivo* validation.

1 Data quality

1.1 TD-NIRS quality parameters

In Fig S1 a), the IRF/phantom box described in the main text (Section 2.1) is shown. If the probe is inserted with the optics facing upwards, the IRF is acquired; otherwise, a phantom measurement is obtained. To monitor the data quality of the TD-NIRS module, the IRF resolution, its shape, and temporal stability must be evaluated through its full-width-half maximum (FWHM) and barycenter. A summary of the quality parameters is presented in Fig S1 b). For clarity, only a portion of the temporal window where the distribution of time-of-flight is reconstructed is highlighted. The TD-NIRS laser is driven at 53 MHz corresponding to an available temporal window of approximately 19 ns wide and a timing resolution of 9.76 ps. For each measurement, one IRF is collected with an integration time of 1 s targeting 10^6 counts per second per wavelength. In Fig S1 c), the results for the FWHM (in ps) and barycenter position in the temporal window (in ns) are reported, with shaded areas indicating the standard deviation calculated over seven months of measurements, for both 685 and 828 nm. For the barycenter here the mean ± standard deviation is reported, alongside the coefficient of variation (CV = standard deviation/mean). For the FWHM only the CV is reported. As mentioned in the main text, the barycenter of the DTOF at 685 nm was 3.9± 0.02 ns (CV = 0.4 %), and at 828 nm it was 3.6 ± 0.02 ns (CV = 0.6 %). The CV of the FWHM was 2.9 % for 685 nm and 1.1 % for 828 nm. In Fig. S2, the results obtained from fitting for μ_a and μ'_s at both 685 and 828 nm, based on the 31 phantom measurements collected in month 3, are reported. Each point in the distribution correspond to the single phantom repetitions (31 measurements x 20 repetitions). In Fig. S3, the effective total hemoglobin (tHb^{ph}) and tissue oxygen saturation ($StO_2{}^{ph}$) extracted are shown. The distributions of the results, obtained by fitting the convolution of the DTOFs acquired with the corresponding IRF of the day (labeled “day” in the figure), were compared to those obtained by convoluting the DTOFs with the first IRF acquired in that specific month (labeled ”month” in the figure), using the Wilcoxon sign-rank test, with significance set at $p<0.05$. A larger CV was found in both μ_a (CV = 2.8%) and μ'_s (CV = 2.5%) for both wavelengths. This translated into a CV of 2.8% and 1.0% in tHb^{ph} and $StO_2{}^{ph}$. However, no statistically significant difference was found when comparing the “day” results to the“month” results for μ_a (p = 0.39 for 685 nm and p = 0.59 for 828 nm) and μ'_s (p = 0.15 for 685 nm and p = 0.19 for 828 nm).

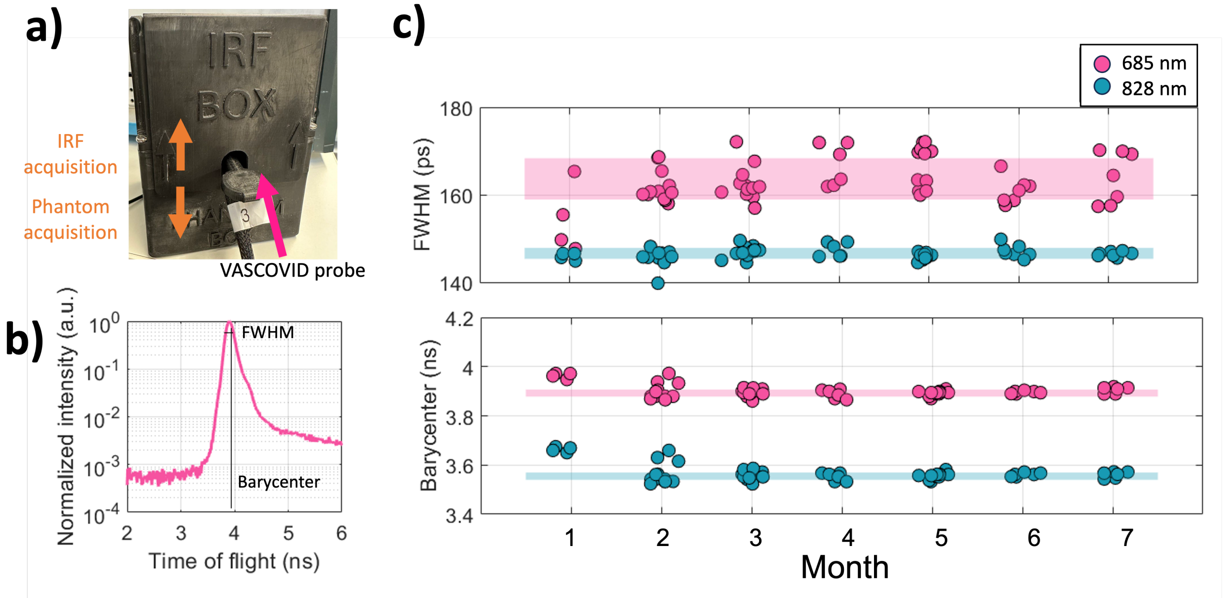

Fig S1: a) smart IRF and phantom box, with the probe optics facing upwards to obtain an IRF. b) An example of an IRF acquired at 685 nm is displayed, with the FWHM and barycenter figure of merit highlighted. c) The graph presents the FWHM and barycenter data for the 59 days of measurements, grouped by month for clarity. The shaded areas represent the standard deviation over all measurements.

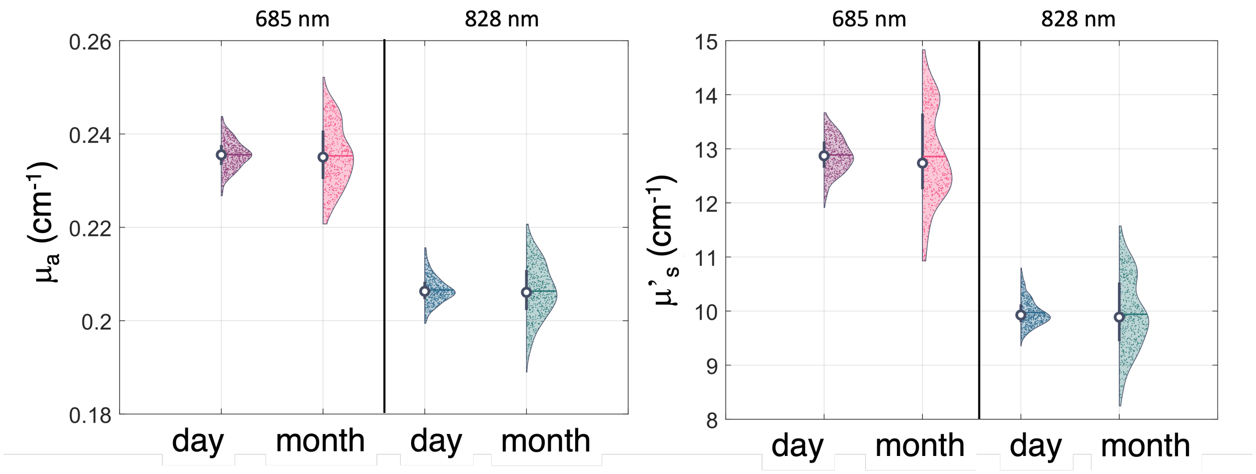

Fig S2: Absorption (μ_a) and reduced scattering (μ'_s) coefficients fitted at 685 and 828 nm for both the day-by-day (day) and month evaluation (month). Each dot in the distribution correspond to a single repetiton in each phantom measurement.

1.2 DCS quality parameters

To assess the performance of the DCS, the count rate (in kHz) and the β parameters are evaluated. An example is depicted in Fig. S4, where the intensity of the detected DCS signal at the baseline of a healthy subject and the β parameter are shown. The β parameter, which depends on the number of modes detected, is calculated as the weighted average of the 2nd to the 4th bin of the

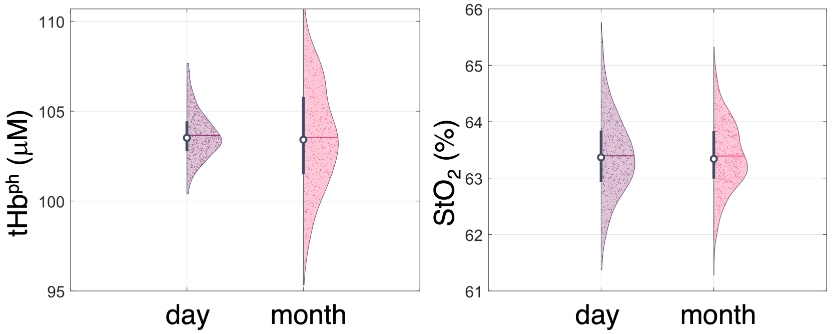

Fig S3: StO_2^{ph} and tHb^{ph} for both the day-by-day (day) and month evaluation (month). Each dot in the distribution correspond to a single repetiton in each phantom measurement.

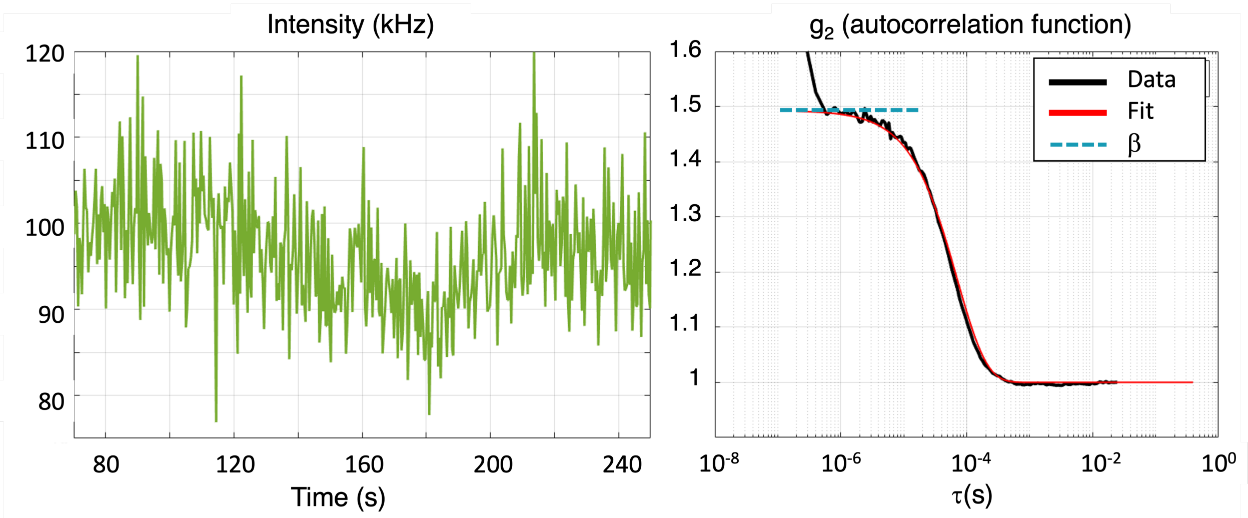

Fig S4: Left panel: intensity recorded during the baseline of a VOT protocol in an healthy subject. The blue shaded area represents the 10 s of integration time. Right panel: intensity autocorrelation function (g_2) as a function of the lag time (τ), averaged over 10s of measurement. The β parameter is calculated as the weighted average of the 2nd to the 4th bin of the $g_2(\tau)$.

autocorrelation function g_2. In this example, a β value of 0.49±0.01 at the baseline is reported. It is possible to observe an increase of g_2 above 1.5 at very short delay times which is attributable to a known detector artifact called afterpulsing. This effect occurs on the nanosecond time scale and is exacerbated under low-SNR conditions, *i.e.*, poor normalization at short delays. Importantly, it does not reflect any physiological signal but results from intrinsic detector and acquisition characteristics. This phenomenon has been well described in the context of diffuse optics even prior to the widespread adoption of DCS in biomedical applications [121,144,145] .

2 Data and information saved

In this section, we report the list of all the variables that are recorded. Each module communicates with the single board computer (SBC) as described in Section 2.1 of the main text and the list of variables are listed and briefly described in Table S1 for the TD-NIRS module, in Table S2 for

the DCS module, in Table S3 for the sensor and safety boards and finally in Table S4 for the PPG module.

Table S1: Data and information stored by the software from the TD-NIRS module. SBC: single board computer; DCS: diffuse correlation spectroscopy.

Variable name	Units	Module origin	Frequency (Hz)	Description
system time TD-NIRS	unix	SBC OS	1	time stamps SBC in the TD-NIRS reference frame
TD-NIRS time	ms	TD-NIRS	1	time stamps TD-NIRS module
DTOF 685	photons	TD-NIRS	1	DTOF at 685 nm
count rate 685	photons/s	TD-NIRS	1	count rate
μ_a 685	1/cm	TD-NIRS	1	real time absorption coeff.
μ'_s 685	1/cm	TD-NIRS	1	real time reduced scattering coeff.
peak position dtof 685	-	TD-NIRS	1	bin corresponding to the peak value of the DTOF at 685 nm
chi2 685	-	TD-NIRS	1	real time chi2 from fitting procedure (fit goodness)
dtof 685 fwhm	-	TD-NIRS	1	full width half maximum for the DTOF at 685
DTOF 828	photons	TD-NIRS	1	DTOF at 828 nm
count rate 828	photons/s	TD-NIRS	1	count rate
μ_a 828	1/cm	TD-NIRS	1	real time absorption coeff.
μ'_s 828	1/cm	TD-NIRS	1	real time reduced scattering coeff.
peak position dtof 828	-	TD-NIRS	1	bin corresponding to the peak value of the DTOF at 828 nm
chi2 828	-	TD-NIRS	1	real time chi2 from fitting procedure (fit goodness)
dtof 828 fwhm	-	TD-NIRS	1	FWHM for the dtof at 828 nm
μ_a 785	1/cm	TD-NIRS	1	real time extrapolated absorption coeff. at 785 nm (See Section)
μ'_s 785	1/cm	TD-NIRS	1	real time extrapolated reduced scattering coeff. at 785 nm (See Section)
HbO	μM	TD-NIRS	1	real time oxy hemoglobin
HbR	μM	TD-NIRS	1	real time deoxy hemoglobin
tHb	μM	TD-NIRS	1	real time total hemoglobin
StO_2	%	TD-NIRS	1	real time tissue oxygen saturation

Variable name	Units	Module origin	Frequency (Hz)	Description
TOE	%	TD-NIRS	1	real time tissue oxygen extration (SpO_2-StO_2)
$MMRO_2$	$\% \cdot cm^2/s$	TD-NIRS	1	real time metabolic rate of oxygen consumption $\approx$ TOE$\cdot$ BFI

Table S2: Data and information stored by the software from the DCS module. SBC: single board computer; DCS: diffuse correlation spectroscopy.

Variable name	Units	Module origin	Frequency (Hz)	Description
system time DCS	unix	SBC OS	38	time stamps SBC in the DCS correlator reference frame
correlator time	ms	DCS	38	time stamps DCS correlator
N channel	-	DCS	38	number of channels available
g2 ch i-th	tau	DCS	38	block containing g_2 and intensity according to N channel
intensity ch i-th	Hz	DCS	38	
BFI	cm^2/s	DCS	38	real time fitted BFI (averaged channels)
Beta	-	DCS	38	real time calculated beta
tau c	s	DCS	38	real time calculated decaying tau (to 1/2 of the g_2)
tau tail	s	DCS	38	real time calculated decaying tau (to 1)
R^2	-	DCS	38	real time calculated R^2 (fit goodness)

Table S3: Data and information stored by the software from the various modules. SBC: single board computer; DCS: diffuse correlation spectroscopy; TD-NIRS: time-domain near-infrared spectroscopy.

Variable name	Units	Module origin	Frequency (Hz)	Description
system time sensors	unix	SBC OS	38	synchronized time stamps
correlator time	ms	DCS	38	
TD-NIRS time	ms	TD-NIRS	38	
touch	-	μCtrl 2	38	touch enabled
acceleration x axis	-		38	acceleration x axis
acceleration y axis	-		38	acceleration y axis
acceleration z axis	-		38	acceleration z axis
acceleration alert	-		38	movement detected
load sensor	-		38	load detected on the sensor
load sensor alert	-		38	high pressure detected
light sensor	-		38	light detection on the photodiode sensor
TD-NIRS lasers alert	-	TD-NIRS	38	ON when TD-NIRS lasers are emitting
DCS laser alert	-	DCS	38	ON when DCS laser is emitting
TD-NIRS quality	-	software	38	it's 1 when quality of the TD-NIRS data is good (fitting and count rate)
DCS quality	-	software	38	it's 1 when quality of the DCS data is good (fitting and count rate)
keypad	-	keypad front panel	38	indicate which key is pressed on the keypad on the front panel
charge battery 1	%	Power management	38	Indicate the charge status of the battery 1
charge battery 2	%		38	Indicate the charge status of the battery 2
charge battery 3	%		38	Indicate the charge status of the battery 3
charge battery 4	%		38	Indicate the charge status of the battery 4
safety board output enabled	-	safety board	38	it's 1 when
reset require	-	safety board	38	it's 1 when the reset button needs to be pressed (after 10 s)

Variable name	Units	Module origin	Frequency (Hz)	Description
spo2	%	pulse-oximeter	38	peripheral oxygenation from pulse oximeter
heart rate	beats/min	pulse-oximeter	38	heart rate from pulse oximeter
pulse-oximeter quality	-	pulse-oximeter	38	
pulse-oximiter info	-	pulse-oximeter	38	
pressure cuff	mmHg	tourniquet	38	
inflate marker	-	software	38	it's 1 when pump is inflated, 0 otherwise
deflate marker	-	software	38	it's 1 when pump is deflating, 0 otherwise
general marker 1	-	keypad/software	38	it's 1 when mark 1 on the keypad is pressed
general marker 2	-	keypad/software	38	it's 1 when mark 2 on the keypad is pressed
quality phase marker	-	software	38	it's 1 when the protocol is in its quality phase (QP)

Table S4: Data and information stored by the software from the SpO_2 module. SBC: single board computer; DCS: diffuse correlation spectroscopy; TD-NIRS: time-domain near infrared spectroscopy.

Variable name	Units	Module origin	Frequency (Hz)	Description
system time PPG	unix	SBC OS	50	
correlator time	ms	DCS	50	synchronized time stamps
TD-NIRS time	ms	TD-NIRS	50	
PPG time	ms	pulse-oximeter	50	time stamps PPG in the pulse-oximeter frame of reference
PPG value	-	pulse-oximeter	50	PPG value

3 Representative reduced scattering time course during a vascular occlusion test

For completeness, a representative μ'_s time course during a VOT is reported in Fig. S5. At both 685 nm and 828 nm, μ'_s remained relatively stable during the whole protocol. However, the wavelength-dependent differences between the two scattering traces might impact oxygenation estimates. As shown in previous work [125], such unaccounted scattering changes can bias CW-NIRS StO_2 by up to 10%, underscoring the advantage of TD-NIRS in separating absorption and scattering contributions.

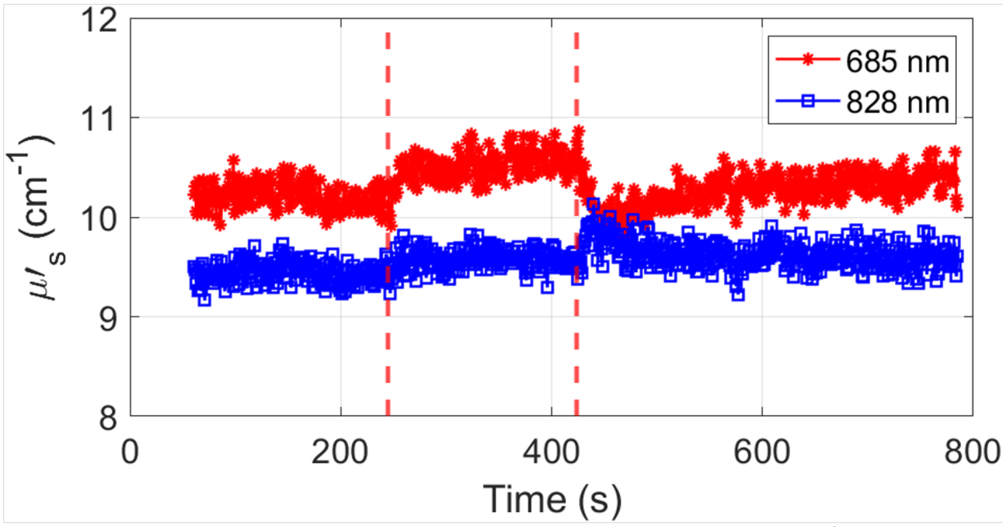

Fig S5: Representative time course of the reduced scattering coefficient (μ'_s) at 685 nm (red) and 828 nm (blue) during a vascular occlusion test. Vertical red dashed lines mark the beginning and end of the occlusion.
